# Complete Mitochondrial Genome of the Poorly Known Fish *Parasinilabeo maculatus* (Cypriniformes: Cyprinidae) and Its Phylogenetic Implications

**DOI:** 10.3390/ani16213447

**Published:** 2026-11-01

**Authors:** Yuting Hu, Guoqing Duan, Huaxing Zhou, Amei Liu, Huan Wang

**Affiliations:** 1 Fishery Institute of Anhui Academy of Agricultural Sciences, Hefei 230031, China; duanguoqing@aaas.org.cn (G.D.); zhouhuaxing@aaas.org.cn (H.Z.); liuamei@aaas.org.cn (A.L.); wanghuan@aaas.org.cn (H.W.); 2 Anhui Province Key Laboratory of Aquaculture & Stock Enhancement, Hefei 230031, China; 3 Agricultural Germplasm Resources Bank of Anhui Province, Hefei 230031, China

**Keywords:** *Parasinilabeo maculatus*, mitochondrial genome, phylogenetic analysis, Semilabeonini, mitogenomics, Cyprinidae

## Abstract

*Parasinilabeo maculatus* is a rare cyprinid endemic to China whose phylogenetic position and taxonomy are poorly understood. We present its first complete mitogenome to clarify its phylogenetic placement within Semilabeonini. The circular mitogenome is 16,606 bp and shows the typical vertebrate gene arrangement: 13 protein-coding genes (PCGs), 22 tRNA genes, 2 rRNA genes, and a control region, with the L-strand replication origin (OL) in the WANCY tRNA cluster. It has a pronounced AT bias (58.7%), with AT-skew (0.094) and GC-skew (−0.250), consistent with other cyprinids. Phylogenetic trees reconstructed using ML and BI methods based on concatenated PCGs show similar topologies: (1) placing *P. maculatus* within Semilabeonini, where it is only provisionally grouped with *P. assimilis* and *P. longicorpus* in the full PCG dataset. This placement receives low gene and site concordance (gCF = 15.38%, sCF = 40.57%) and conflicts with the first + second codon position tree, in which *P. maculatus* is sister to *Hongshuia microstomata* rather than to its congeners; (2) supporting the monophyly of Semilabeonini and of several genera (e.g., *Ptychidio*), whereas the monophyly of *Parasinilabeo* is tentatively resolved at best and is therefore treated here as tentative (see point 1); (3) preliminarily revealing non-monophyly of some genera (e.g., *Sinocrossocheilus*) based on the current sampling, suggesting that some relationships may not fully match morphological classifications. However, these inferences rest on mtPCGs and limited taxon sampling and require testing with broader taxonomic and genomic sampling. These mitogenomic data provide a genomic resource for taxonomic and conservation studies of Semilabeonini.

## Simple Summary

The genus *Parasinilabeo* contains small freshwater fishes endemic to China. One species, *Parasinilabeo maculatus*, is very rare and poorly studied owing to its very limited distribution and seasonal habitat shifts reported by local residents. Understanding its genetic makeup is crucial for its conservation. Here, we characterized its complete mitogenome for the first time; it shows typical vertebrate structural features. We also constructed phylogenetic trees from concatenated PCGs using ML and BI. Our trees consistently support the monophyly of Semilabeonini (Labeoninae). In the full PCG dataset, *P. maculatus* is provisionally placed with *P. assimilis* and *P. longicorpus*. This placement is not robust, however: concordance factors are low, and it conflicts with the first + second codon position tree, in which *P. maculatus* is sister to *Hongshuia microstomata*. Accordingly, *Parasinilabeo* is monophyletic only in the full PCG dataset and, being likewise not robust, is treated here as tentative. In contrast, several genera (e.g., *Ptychidio*) are robustly monophyletic. Our trees also preliminarily suggest non-monophyly in some genera (e.g., *Sinocrossocheilus*, *Pseudocrossocheilus*), each represented by few species. These mtPCG-based results tentatively suggest that some relationships do not fully match morphological classifications, pending testing with broader taxonomic and genomic sampling. This work provides essential genetic information for understanding Semilabeonini biodiversity and conserving this rare species.

## 1. Introduction

Semilabeonini is a tribe-level taxon within the subfamily Labeoninae (Cypriniformes: Cyprinidae). The lineage was initially proposed as the informal clade Semilabeoina by Yang et al. (2012) [1] and was later treated by Kottelat (2025) [2] as a subtribe within Labeonini. Here, we adopt Labeoninae as the subfamily-level taxon and use Semilabeonini as the tribe-level name throughout. In authoritative databases such as UniProt and NCBI, this lineage is treated as a tribe comprising at least 19 genera. Recent molecular phylogenetic analyses have repeatedly recovered a major clade within Labeoninae (Clade IV) that includes *Ageneiogarra*, *Decorus*, *Discogobio*, *Parasinilabeo*, and their close relatives [3,4,5]; this clade corresponds to Semilabeonini in the broad sense [6].

Semilabeonini is distributed mainly in southern China and Southeast Asia, with a particular concentration in karst regions [7]. This tribe exhibits a remarkable diversity in oromandibular structures—modified lips, barbels, and rostral folds, which have traditionally been used for generic diagnosis [6,7,8]. However, molecular evidence indicates that these traits arose repeatedly in independent lineages as convergent adaptations to fast-flowing habitats, rather than reflecting shared ancestry [2,6,8,9]; even the oral disc is not homologous across lineages [2]. Such pervasive homoplasy undermines morphology-based classifications and obscures generic limits within Semilabeonini, underscoring the need for molecular phylogenetic analyses [6]. Despite previous molecular efforts [3,4,7], the internal phylogenetic relationships of this tribe remain unresolved, mainly owing to insufficient taxon sampling.

Within Semilabeonini, the genus *Parasinilabeo* Wu, 1939 is endemic to China and restricted to tributaries of the middle and lower reaches of both the Yangtze and the Pearl River systems [10,11,12,13,14,15]. Its taxonomic validity was formally established through the fixation of its type species (*P. assimilis*) by Kottelat and Zhang (2003) [16]. The genus currently contains six recognized species (FishBase: https://www.fishbase.org, accessed on 14 September 2026), all small-bodied and typically inhabiting rapid-flowing waters [10,11,12,13,14,15]. As in other Semilabeonini, *Parasinilabeo* species possess highly specialized lips and associated structures, but the pervasive homoplasy discussed above casts doubt on the diagnostic value of these characters [7,10,11,12,13,14,15,17,18]. Consequently, the monophyly of *Parasinilabeo* remains uncertain [7,8,9]. Recent molecular evidence further suggests that *P. longicorpus* might be a synonym of *P. assimilis* [7]. To date, molecular work on *Parasinilabeo* has largely relied on a few mitochondrial or nuclear genes, and complete mitogenomes are available for only two species [19,20]. Key genomic features, such as codon usage bias, tRNA secondary structures, and control region motifs, remain largely unexplored.

**Figure 1:**
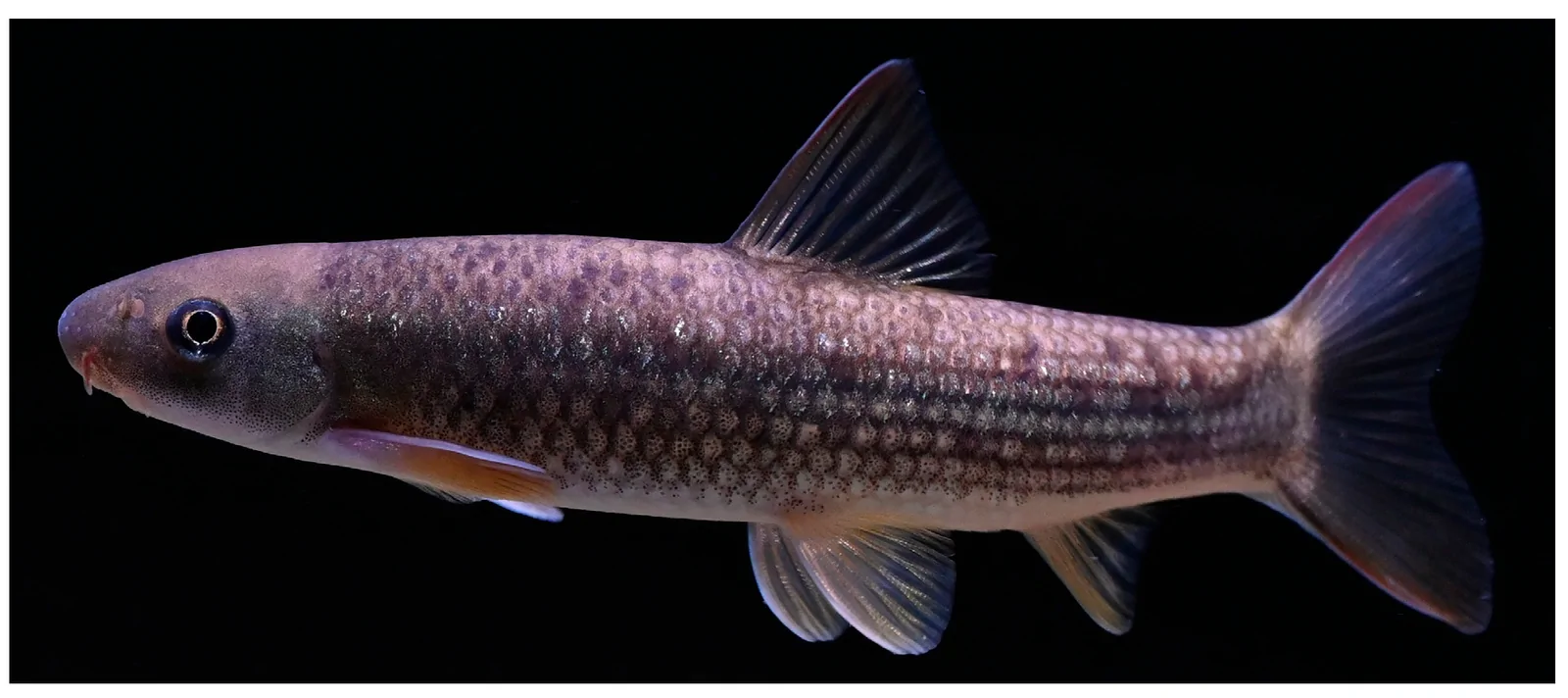
*Parasinilabeo maculatus*, wild-caught live specimen showing natural coloration.

Within this genus, *P. maculatus* is among the most poorly studied rare freshwater fishes in China, with no molecular data currently available. It was originally described by Zhang (2000) from the Qiupu River in Shitai County, Anhui Province, China [12] (Figure 1). More than two decades after its discovery, research on this species has remained scarce: fundamental biological and ecological information is virtually undocumented, including population size, life-history traits, habitat preferences, reproductive behavior, and demographic trends. This gap largely reflects the species’ rarity and extremely restricted distribution. To date, the few collection records are confined to its type locality and three small tributaries in the upper reaches of the Ganjiang River, Jiangxi Province [21,22]; the Ganjiang River is a major tributary of the middle Yangtze, entering it via Poyang Lake. Population densities also remain generally low. Such range restriction and low density may render the species particularly vulnerable to genetic drift and habitat degradation.

Notably, local residents reported a seasonal habitat shift during our survey. From October to April, the species retreats into deep pools, rocky crevices, and subterranean rivers, becoming largely inaccessible to conventional sampling. From May to September, it appears briefly in torrential, well-oxygenated riffles or gravel-bedded reaches—the only window for observation and collection.

Similar seasonal behavior has been reported in the congeneric *P. longicorpus* and *P. longibarbus* [14,15]. Together with habitat fragmentation, this locally reported winter-retreat and summer-emergence behavior has hampered in-depth investigations into its population demography, reproductive ecology, and genetic diversity. This behavior has not been quantitatively verified in the present study.

To address these knowledge gaps, molecular data for *P. maculatus* are urgently needed. Because of its maternal inheritance, moderate evolutionary rate, and conserved structural architecture, the mitogenome is a powerful tool for phylogenetic reconstruction and population genetic analyses, and a practical means of generating foundational genetic data for this elusive species. However, the complete mitogenome of *P. maculatus* remains uncharacterized. Here, we therefore sequenced and annotated its complete mitogenome with three objectives: (i) to characterize the structure and organization of the mitogenome, including tRNA secondary structures and control region features; (ii) to examine codon usage patterns in *P. maculatus* and selection pressures relative to closely related species within Semilabeonini; (iii) to infer the phylogenetic position of *P. maculatus*. This mitogenome represents a reference sequence for *P. maculatus* that can be incorporated into future phylogenetic, population genetic, and conservation studies.

## 2. Materials and Methods

### 2.1. Specimen Collection and Initial Processing

Specimens were collected on 1 June 2026 from the type locality (30.117° N, 117.641° E): Qiupu River in Shitai County, Anhui Province, China, in compliance with applicable national laws and regulations. A special fishing permit (Wan Yu Zhuan [2026] No. 46) was issued by the Department of Agriculture and Rural Affairs of Anhui Province for this collection. To balance resource conservation with future research needs, live individuals suitable for artificial domestication and controlled breeding were targeted for sampling. Nine live specimens were captured using cage traps deployed along the riverbed and immediately transferred to indoor rearing tanks simulating the natural hydrological and physicochemical conditions of the native habitat (water temperature, flow velocity, dissolved oxygen, and substrate composition). During acclimation, one individual died unexpectedly. To maximize its scientific utility, a muscle tissue sample was excised from the right dorsal region and preserved in absolute ethanol for molecular analyses, and the remainder was fixed in 4% formalin as a permanent voucher for morphological examination. Molecular analysis therefore relies on a single individual (*n* = 1), as sampling was limited to the naturally deceased specimen, so that the remaining individuals could be kept alive for artificial domestication and breeding. Species-level identification followed the morphological criteria of the protologue. All voucher materials were deposited in the ichthyological collection maintained by the Fishery Institute of the Anhui Academy of Agricultural Sciences (Voucher number: AAAS20260601Pm1).

### 2.2. Genomic DNA Isolation and Library Construction

Total genomic DNA was purified from approximately 200 mg of muscle tissue dissected from a single adult *P. maculatus* using the Ezup Column Animal Genomic DNA Purification Kit (B518251, Sangon Biotech, Shanghai, China), with all procedures conducted strictly according to the manufacturer’s instructions. DNA concentration was determined via fluorometric quantification using the Qubit dsDNA HS assay kit (Thermo Fisher Scientific, Waltham, MA, USA), and sample integrity was assessed by electrophoresis on a 1% agarose gel. An aliquot of 500 ng of purified, high-molecular-weight DNA was subjected to mechanical shearing with a Covaris M220 instrument (Covaris, Woburn, MA, USA), targeting an average fragment size of 400 bp. Library preparation was performed using the Hieff NGS^®^ MaxUp II Kit (YEASEN, Shanghai, China) following a standard Illumina-compatible workflow, including end-repair, A-tailing, adapter ligation, and limited-cycle PCR enrichment. After a size-selection step to recover fragments centered around 400 bp, the molarity of the final library and fragment length distribution were verified using a Qubit 4.0 fluorometer (Thermo Fisher Scientific, Waltham, MA, USA) and 2% agarose gel electrophoresis. The multiplexed library was then subjected to paired-end sequencing (150 bp read length) on the DNBSEQ-T7 platform (BGI, Shenzhen, China).

### 2.3. Mitogenome Assembly, Annotation and Confirmation

Raw sequencing reads (Table S1) were initially filtered and quality-trimmed with Fastp 0.11.2 [23]. A total of 58,831,532 raw reads were generated, yielding 58,606,172 clean reads (99.62%). The clean data (Table S1) were first assembled into contiguous sequences using SPAdes 3.15 [24] with default parameters, and the complete circular mitogenome was subsequently reconstructed using the MITObim 1.9.1 iterative mapping approach [25], using the mitogenome of *P. assimilis* (NC025947) as the initial reference (Table S2). Gene boundaries and functional elements were predicted using MITOS 2.1.7 [26], followed by manual refinement in Geneious Prime 2025.1 (https://www.geneious.com, accessed on 24 July 2026). Start and stop codons were verified manually by comparison with the mitogenomes of closely related Labeoninae species [19,20], and annotations were checked against the NCBI reference sequences. A circular gene map of the assembled genome was generated with MitoDraw module in MitoFish (v2026.09; https://mitofish.aori.u-tokyo.ac.jp, accessed on 8 September 2026) [27]. Assembly validation was performed by PCR amplification and bidirectional Sanger sequencing of the cytochrome *b* (*cytb*) gene and D-loop using published Cypriniformes-specific primers (Table S3) [28,29]. PCR conditions followed those described in [28,29]. The expected amplicon sizes were 1235 bp for *cytb* and 1035 bp for the D-loop, and each target was independently amplified in two replicate reactions. Sequence alignments in MEGA 12.09 [30] showed that the Sanger sequences of both amplicons were 100% identical to the corresponding regions of the assembled mitogenome.

### 2.4. Evolutionary Constraints and Sequence Divergence Analysis

The nucleotide composition, AT-skew and GC-skew values, codon usage bias, and relative synonymous codon usage (RSCU) of the 13 protein-coding genes (PCGs) were calculated using PhyloSuite 2 [31], with stop codons treated as a synonymous group in the RSCU analysis. The secondary structure of tRNAs was predicted using tRNAscan-SE 2.0 (https://trna.ucsc.edu/tRNAscan-SE/, accessed on 30 August 2026) and visualized using Forna (http://rna.tbi.univie.ac.at/forna, accessed on 31 August 2026). The D-loop structure was identified following the general identification criteria for fish control regions [29] and by alignment with homologous sequences from 48 Semilabeonini species (Table 1) and from *Labeo fimbriatus* [32], for which the control-region elements have been explicitly delimited. Conserved motifs were assigned on this basis.

Nonsynonymous (Ka) and synonymous (Ks) substitution and Ka/Ks ratios were calculated using the Nei–Gojobori method implemented in DnaSP 6.0 [33] with default parameters to evaluate selective pressures during PCG evolution among Semilabeonini (Table 1). Identical sequence pairs yielding Ks = 0 were excluded from the gene-wise estimates, as such comparisons produce undefined Ka/Ks values. Each PCG was extracted as described in Section 2.5, with stop codons excluded. Ka and Ks were estimated for each pairwise sequence comparison; the mean Ka and mean Ks were then calculated across all pairwise comparisons among the 48 Semilabeonini sequences, and Ka/Ks was obtained as the ratio of these two means.

Interspecific and intraspecific genetic divergences within each genus of Semilabeonini (Table 1) based on concatenated PCGs (stop codons excluded), expressed as Kimura two-parameter (K2P) distances, were estimated using MEGA 12.09 [30]. Genus-level averages were calculated as follows: (1) sequences with uncertain species assignments were excluded; (2) for species with multiple sequences, the NC (reference) sequence was preferred when available; and (3) for *Discogobio bismargaritus*, no NC (reference) sequence was available, so PQ789988 was selected. These PCG sequences were processed as described in Section 2.5. To compare intraspecific and interspecific genetic distances within *Discogobio* (including *Discocheilus wui* and *Discolabeo wuluoheensis*), these values were sorted in ascending order using Excel and visualized as a scatter plot. Substitution saturation was assessed using Xia’s test [34] implemented in DAMBE7 [35], with the observed Iss compared against the critical value Iss.c under both symmetrical and asymmetrical topology assumptions. The analysis was performed separately on the full concatenated PCG dataset, on the first and second codon positions, and on the third codon position.

### 2.5. Taxon Sampling and Phylogenetic Analysis

All data processing for phylogenetic analyses was performed in PhyloSuite 2 [31]. We downloaded all publicly available mitogenomes of Semilabeonini from GenBank and removed identical DNA sequences, retaining only one copy per unique sequence, with priority given to NC (reference) accessions; retained sequences were treated as independent terminals, as individual- and population-level metadata are generally lacking in GenBank records. The final dataset comprised 50 mitogenomes: 48 sequences from 18 genera of Semilabeonini and two species (*Spinibarbus sinensis* and *Tor sinensis*) of Barbinae as outgroups (Table 1). All PCGs were extracted and aligned individually using MAFFT 7.520 [36] with default parameters, and then concatenated into a single matrix (11,382 bp) (stop codons excluded). For the concatenated PCGs, optimal partitioning schemes and substitution models (comprising 7 partitions; Table S4) were selected under the Bayesian Information Criterion (BIC) using ModelFinder 2.2.0 [37], with each gene and each codon position treated as a separate potential block. Phylogenetic trees were reconstructed using both maximum likelihood (ML) and Bayesian inference (BI). ML analysis was conducted in IQ-TREE 3.01 [38] using the standard nonparametric bootstrap with 1000 replicates. BI analysis was performed in MrBayes 3.2.6 [39] with two independent runs of 10 million generations, sampling every 1000 generations and discarding the first 25% as burn-in. Convergence was confirmed by an average standard deviation of split frequencies (ASDSF) that remained stably below 0.01, Potential Scale Reduction Factor (PSRF) approaching 1.0, and Effective Sample Size (ESS) > 100 for all parameters (Table S5). All resulting trees were visualized and annotated using the Interactive Tree of Life (iTOL) 7.6 (https://itol.embl.de/, accessed on 14 September 2026).

To assess the potential effect of third-position saturation, trees were additionally inferred from first and second codon positions only. These trees were inferred with the same ML pipeline (IQ-TREE 3.01, 1000 ultrafast bootstrap replicates and SH-aLRT test), using the same partitioning scheme adapted to the reduced dataset; no BI analysis was performed on this reduced dataset. Gene and site concordance factors (gCF and sCF) were calculated in IQ-TREE 3.01 [38] for each node of the ML tree inferred from the full concatenated PCG dataset, using the 13 single-gene trees and the concatenated alignment, with 1000 quartets for sCF.

## 3. Results

### 3.1. Mitochondrial Structural Characteristics

**Figure 2:**
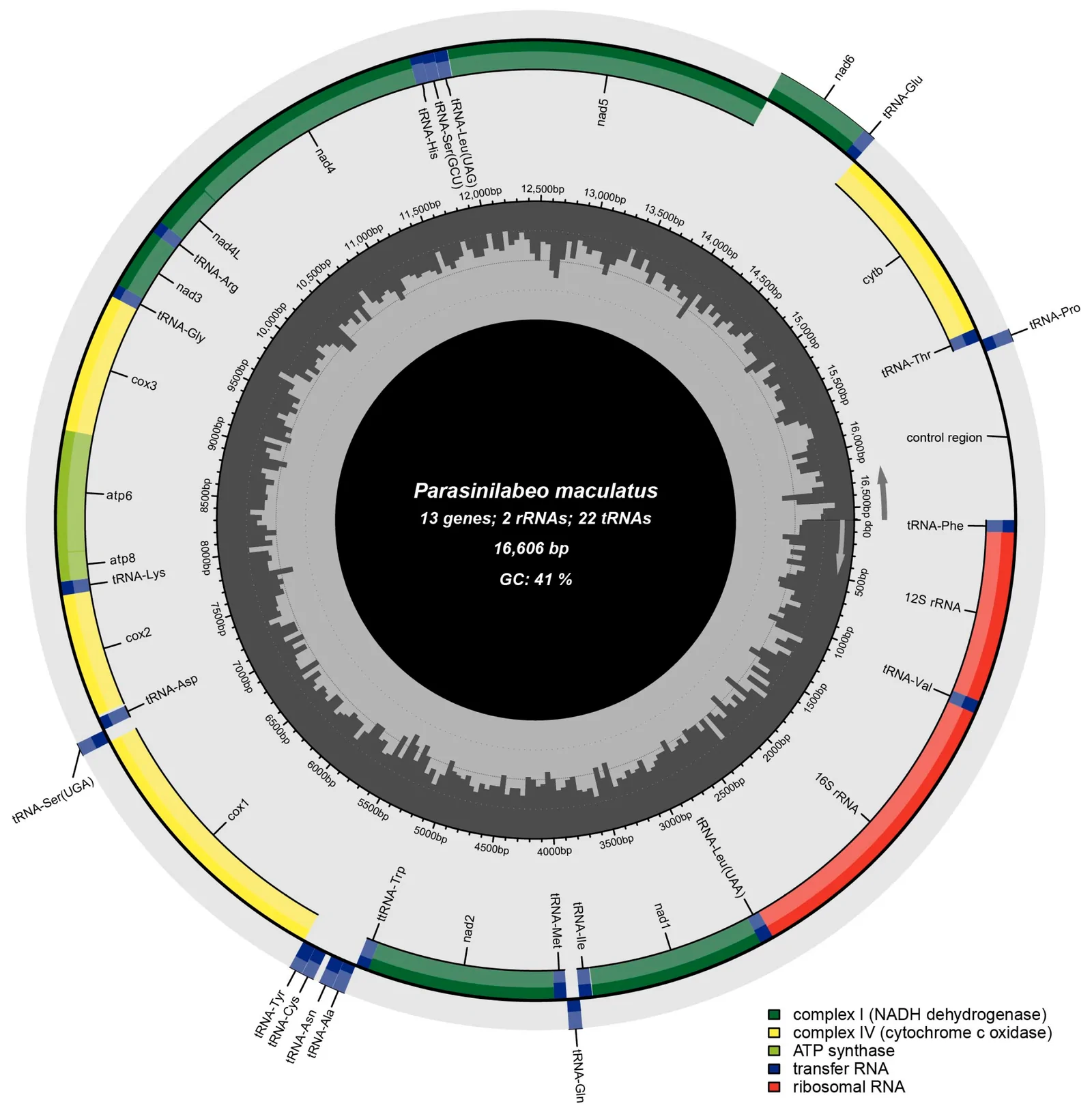
Circular map of the mitogenome of *P. maculatus*. Two tracks (from inside out): GC content and genes. In the outermost gene track, inner arcs indicate genes on the Light strand, and outer arcs indicate genes on the Heavy strand. The arrow represents the replication direction of the light strand.

The assembled complete mitogenome of *P. maculatus* (GenBank: PZ785808) was 16,606 bp in length, with a mean sequencing depth of 980.4× and 100% coverage. A total of 157,046 reads (0.27% of the clean reads) were used for assembly (Table S2). The mitogenome exhibits a circular double-stranded structure characteristic of vertebrate mitogenomes (Figure 2, Table 2). Annotation identified the typical mitochondrial gene repertoire of 37 genes, comprising 22 tRNA, 13 PCGs, two rRNAs, and a single control region (D-loop), similar to the other two *Parasinilabeo* species [19,20]. Regarding strand occupancy, nine genes—*tRNA-Gln*, *tRNA-Pro*, *tRNA-Glu*, *nad6*, *tRNA-Ser*^(UGA)^, *tRNA-Tyr*, *tRNA-Cys*, *tRNA-Asn*, and *tRNA-Ala*—are localized on the light strand (L strand), while the heavy strand (H strand) carries the remainder. The gene order and composition are conserved and conform to the canonical arrangement of most teleost mitogenomes; no gene rearrangements or duplications were detected.

Twelve PCGs utilize the standard vertebrate start codon (ATG), while *cox1* begins with GTG. Seven PCGs (*cox1*, *atp8*, *atp6, nad1*, *nad4L, nad5,* and *nad6*) terminate with complete stop codons (TAA or TAG), whereas the remaining PCGs use an incomplete termination codon (T-- or TA-), a common feature in metazoan mitogenomes. The 22 tRNA genes, ranging from 66 to 76 bp, exhibit typical cloverleaf secondary structures, with the exception of *tRNA-Ser*^(GCU)^, which lacks the dihydrouridine (DHU) arm—a feature commonly observed in vertebrate mitogenomes (Figure S1) [40].

The 945-bp D-loop (15,662–16,606) can be divided into three major domains: an extended termination-associated sequence (ETAS) domain (15,662–15,905) containing a TAS motif, a central conserved domain (CD) (15,906–16,228) with three conserved motifs (CSB-F, CSB-E, CSB-D), and a conserved sequence block (CSB) domain (16,229–16,606) containing the CSB-1, CSB-2, CSB-3 motifs and AT repeat motifs. The positions of each element are listed in the note to Table 2 and illustrated in Figure S2; the conservation of these motifs is supported by sequence logos shown in Figure S3. The AT repeat region consists of variable copy numbers of an AT dinucleotide unit, ranging from 10 to 54 copies among the examined species, with a consensus repeat unit of (AT)n. In *P. maculatus*, this region comprises 13 copies (26 bp). Because of the variable copy number and the resulting length heterogeneity, this region was not included in the sequence logo analysis.

A total of six gene overlaps were identified, with the largest overlap of 7 bp occurring between *atp8* and *atp6*, as well as between *nad4L* and *nad4*. Additionally, 13 intergenic spacers were observed. The largest one spans 33 bp and is located between *tRNA-Asn* and *tRNA-Cys*, where a stem-loop (OL) was identified (Figure S4). Overall, the genome structure of *P. maculatus* conforms to the typical vertebrate mitochondrial architecture, with no unusual features detected.

### 3.2. Nucleotide Composition and Skewness

The *P. maculatus* mitogenome was AT biased (58.7%), with a marked anti-G bias (15.5%) (Table 3), a commonly observed feature in teleost mitogenomes [41]. The overall base composition yielded a positive AT-skew (0.094) and a negative GC-skew (−0.250). The concatenated 13 PCGs (11,403 bp, including stop codons) showed similar AT content (59.0%). The H-strand, encoding 12 of the 13 PCGs, exhibited a positive AT-skew and a negative GC-skew, whereas the sole L-strand-encoded PCG (*nad6*) showed a strikingly different profile, with elevated T and G content, a strongly negative AT-skew and positive GC-skew, consistent with other teleost mitogenomes [41].

Pronounced compositional heterogeneity was evident across codon positions (Table 3). First positions showed a balanced nucleotide distribution with slight positive skews, second positions were AT-biased with negative skews, and third positions were the most AT-rich, with a strong positive AT-skew and negative GC-skew, mainly owing to high A and low G content on the H-strand.

Among H-strand PCGs, AT content ranged from 52.5% (*nad4L*) to 61.9% (*atp8*), and showed negative GC-skews, being lowest in *nad2*, *nad4L*, and *nad4* (Table 3). AT-skew values were more variable, being highest in *nad2* and *atp8*, while *cox1* and *nad3* were negative. The L-strand-encoded *nad6* showed distinct skewness.

The two rRNAs were AT-rich with positive AT-skews and negative GC-skews (Table 3). tRNAs displayed moderate AT content and near-neutral skews, and the D-loop was the most AT-rich region (67.1% AT) with slightly negative GC-skew and near-neutral AT-skew.

### 3.3. Codon Usage Patterns

**Figure 3:**
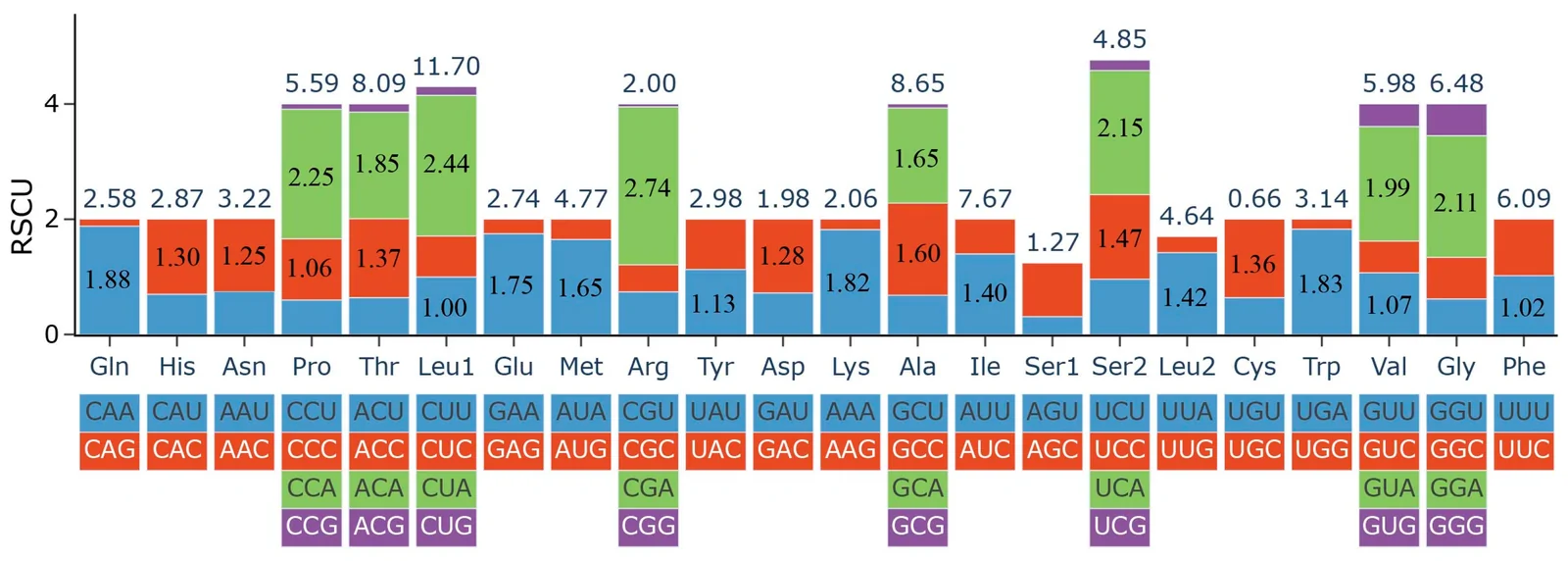
Amino acid composition and codon usage bias of the 13 PCGs in the *P. maculatus* mitogenome. The upper panel shows the percentage of each amino acid relative to the total amino acids encoded by the 13 PCGs (stop codons excluded). The inner panel shows the relative synonymous codon usage (RSCU) values, with only overrepresented codons (RSCU > 1.0) displayed. Detailed codon counts and RSCU values are listed in Table 4.

Codon usage analysis of the 13 PCGs revealed a strong preference for A/U-ending codons (Figure 3, Table 4). The PCGs encoded a total of 3794 amino acids (excluding stop codons), corresponding to 3794 codons. Among these amino acids, leucine (Leu1 + Leu2 = 16.34%) was the most abundant amino acid, whereas cysteine (0.66%) was the least represented (Figure 3, Table 4). RSCU calculations identified 27 overrepresented codons (RSCU > 1.0), most ending in A or U, although several C-ending codons (e.g., ACC, CCC) also exceeded 1.0 (Figure 3). Four codons exhibited RSCU values exceeding 2.0: CGA (Arg, 2.74), CUA (Leu, 2.44), CCA (Pro, 2.25), and UCA (Ser, 2.15), indicating a strong A-bias at the wobble position. Furthermore, under the vertebrate mitochondrial genetic code, UGA was translated as tryptophan and was strongly favored (RSCU = 1.83) over its synonymous counterpart UGG (RSCU = 0.17). UAA was overwhelmingly dominant (RSCU = 3.43), whereas UAG occurred only rarely (RSCU = 0.57), and both AGA and AGG were completely absent (RSCU = 0.00). Overall, the predominance of A/U-ending codons is consistent with the AT-rich base composition of the *P. maculatus* mitogenome.

### 3.4. Evolutionary Dynamics and Genetic Divergence

**Figure 4:**
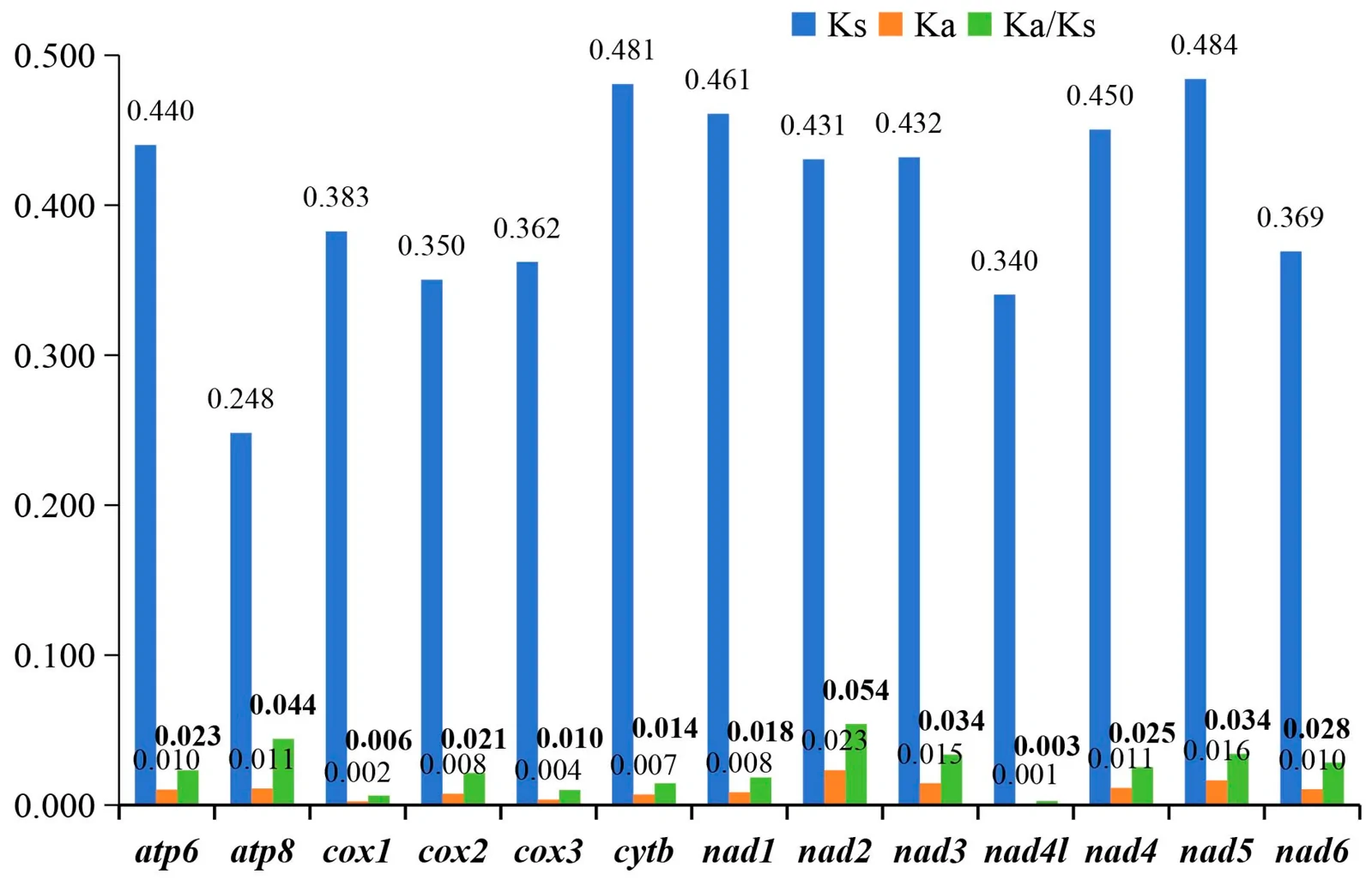
Synonymous substitution rate (Ks), nonsynonymous substitution rate (Ka), and their ratio (Ka/Ks) of the 13 protein-coding genes (PCGs) in Semilabeonini. Ka/Ks is the ratio of mean Ka to mean Ks across all pairwise comparisons among the 48 Semilabeonini sequences. Bold values indicate Ka/Ks values.

The Ka/Ks ratios of all PCGs from Semilabeonini were far below 1 (Figure 4), ranging from 0.003 (*nad4L*) to 0.054 (*nad2*). Ks values ranged from 0.248 (*atp8*) to 0.484 (*nad5*), while Ka values ranged from 0.001 (*nad4L*) to 0.023 (*nad2*); Ka was consistently much lower than Ks. Comparisons among genes revealed that selective constraints were not uniform: (1) except for *nad4L, cox1* and *cox3* exhibited the lowest values (0.006 and 0.010, respectively), indicating that cox genes are generally under purifying selection overall; (2) substantial variation was observed within nad genes: *nad4L* was extremely low (0.003), whereas *nad2*–*nad6* (0.025–0.054) were all markedly higher than cox genes and *cytb* (0.014), suggesting that selective constraints differ among complex I subunits (NADH dehydrogenase); (3) *atp8* showed a relatively high Ka/Ks (0.044), but its Ks (0.248) was the lowest among all genes and its Ka (0.011) was not elevated, likely reflecting statistical effects related to the short gene length and scarcity of synonymous sites.

Genetic distance analysis within each genus based on PCGs revealed some abnormal values (Table S6): (1) exceptionally low interspecific distance (<1.00%), 0.04–0.88% among *Discogobio bismargaritus* PP384505, *Discogobio laticeps* NC045918, and *Discogobio yunnanensis* NC025319, and 0.35% between *Semilabeo notabilis* NC045916 and *S. obscurus* MN229747, falling within the range typically expected for intraspecific variation in fishes [42]; and (2) exceptionally high intraspecific distance, e.g., 4.25% between two *Discogobio macrophysallidos* sequences, exceeding the average interspecies value (4.10%) within the genus. However, PCG-based distances among the *Parasinilabeo* species ranged from 7.47% to 9.62% (Table S6), indicating substantial mitochondrial divergence. This level of divergence is comparable to other interspecific distances in the genus.

Additionally, a scatter diagram (Figure S5) of genetic distance of *Discogobio* (including *Discocheilus wui* and *Discolabeo wuluoheensis*) showed that (i) interspecific distances within *Discogobio* are generally low to moderate (≤7.15%, averaging 4.10%); (ii) distances between *Discogobio* and *Discocheilus wui*, and between *Discogobio* and *Discolabeo wuluoheensis* also fall within this range; and (iii) distances between three uncertain species and other sequences vary widely (0.19–7.17%), suggesting they may comprise multiple species, including both sampled and unsampled species.

### 3.5. Phylogenetic Reconstruction of Semilabeonini

**Figure 5:**
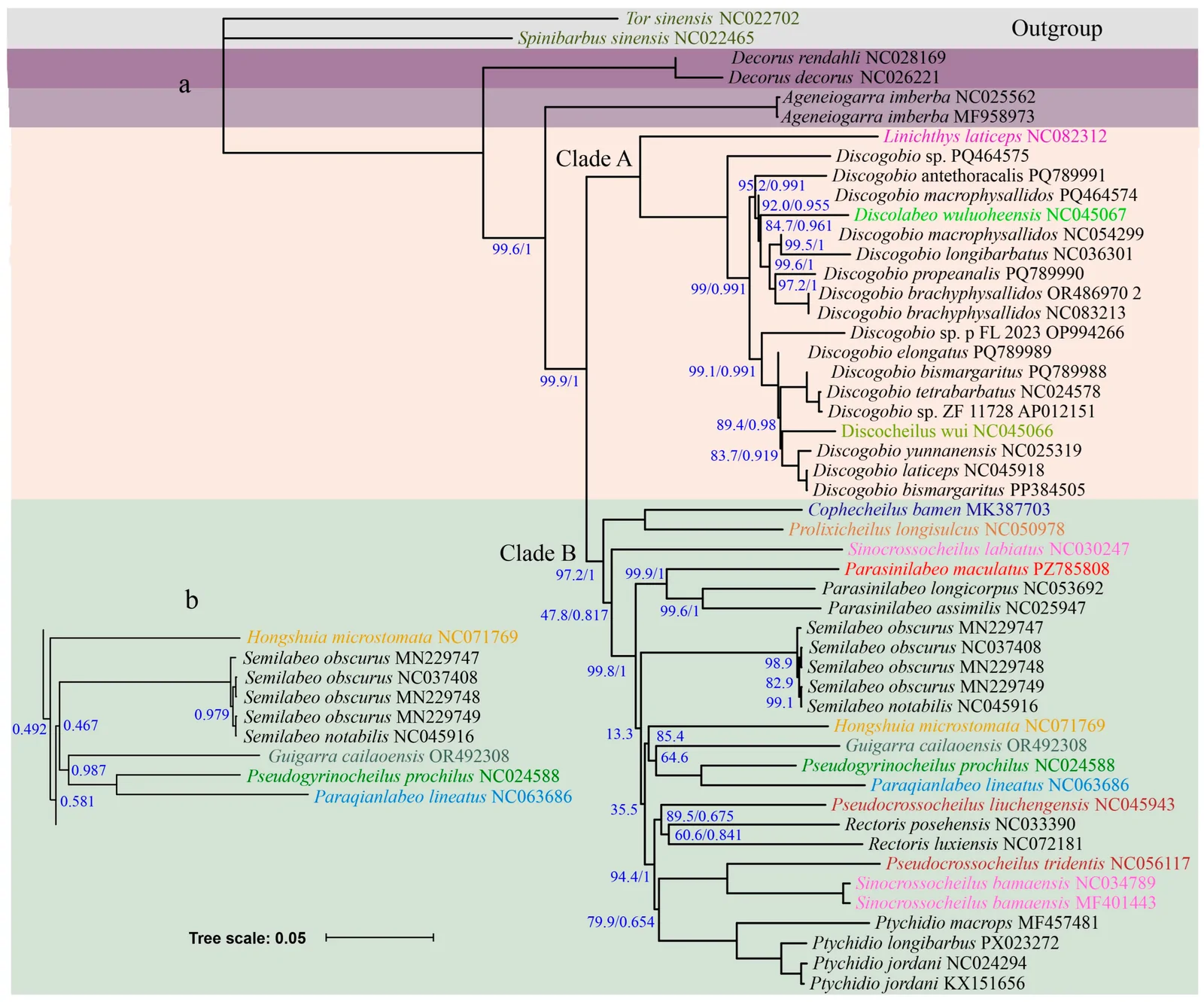
Phylogenetic tree reconstructed using maximum-likelihood (ML) (**a**) and Bayesian inference (BI) methods (**b**) based on concatenated 13 PCGs from 48 Semilabeonini species and two outgroups. Node values: bootstrap support (BS, left) and posterior probability (PP, right); unlabelled nodes indicate maximal support (100%/1.00). The target species is highlighted in red, and its position is provisionally placed within *Parasinilabeo*; see Section 3.5 for the low gene and site concordance factors and the conflict with the first + second codon position tree (Figure S6).

The ML and BI analyses recovered largely congruent topologies (Figure 5a,b), differing only in the relative positions of *Semilabeo* and *Hongshuia microstomata* with low nodal support (BS < 50%, PP < 0.60). In addition, the ML tree solely based on the first and second codon positions was largely congruent with the full-matrix topology, except at several deep nodes. Most notably, the position of *P. maculatus* conflicts between the two datasets: it was provisionally placed as sister to *H. microstomata* in the first + second codon position tree, rather than with its congeners as in the full PCG tree (Figure S6). In this reduced dataset, the node uniting *P. maculatus* with *H. microstomata* received SH-aLRT = 97.3% and UFBoot = 92% (Figure S6).

Semilabeonini was recovered as monophyletic relative to the two Barbinae outgroups (BS = 100%, PP = 1). Within Semilabeonini, the two *Decorus* species occupied the basalmost position with maximal support (BS = 100%, PP = 1), followed by *Ageneiogarra imberba* (BS = 99.6%, PP = 1). The remaining taxa were resolved into two major clades (Clade A and Clade B).

Clade A comprised four genera—*Discocheilus*, *Discogobio*, *Discolabeo*, and *Linichthys*; *Linichthys laticeps* was placed as sister to the *Discocheilus* + *Discogobio* + *Discolabeo* clade (BS = 100%, PP = 1).

Clade B contained all remaining genera; within it, *Semilabeo* and *Ptychidio* were each recovered as monophyletic in the full PCG dataset (BS ≥ 98.9%, PP = 1), whereas *Rectoris* received lower support (BS = 60.6%, PP = 0.841). However, the monophyly of *Parasinilabeo* is not robust, and *P. maculatus* is only provisionally placed with *P. assimilis* and *P. longicorpus*: *P. maculatus* falls outside the genus when third codon positions are excluded and is instead sister to *H. microstomata* (Figure S6), and the node uniting it with *P. assimilis* and *P. longicorpus* also has low concordance (gCF = 15.38%, sCF = 40.57%; Section 3.6). Accordingly, we treat the monophyly of *Parasinilabeo* as tentatively resolved at best, and as tentative here. Nevertheless, in the full PCG dataset, *P. maculatus* was provisionally placed as sister to the *P. assimilis* + *P. longicorpus* group (BS = 99.9%, PP = 1). Based on the present mitochondrial dataset, three genera were not recovered as monophyletic. *Discogobio* (14 species/lineages sampled; Table 1) was not monophyletic because *Discocheilus wui* and *Discolabeo wuluoheensis* were nested within it, although most sampled species formed a large, well-supported subclade (BS = 99%, PP = 0.991). *Pseudocrossocheilus* and *Sinocrossocheilus*, each represented by only two species (*P. liuchengensis* and *P. tridentis*; *S. bamaensis* and *S. labiatus*), were likewise not recovered as monophyletic. Relationships among the deeper nodes within Clade B were generally weakly supported (BS < 70%, PP < 0.95). Given these sampling and support limitations, the non-monophyly of *Pseudocrossocheilus* and *Sinocrossocheilus* should be regarded as preliminary.

### 3.6. Substitution Saturation and Concordance Factors

Concordance factors were calculated for each node of the ML tree inferred from the full concatenated PCG dataset (Figure 5a; Table S8). Substitution saturation analysis showed that Iss was significantly lower than Iss.c for all three datasets (full: Iss = 0.116; pos 1 + 2: Iss = 0.034; pos 3: Iss = 0.317; all *p* < 0.0001; Table S7), indicating little saturation overall.

Concordance factors were calculated for each node (Table S8). The deep nodes separating the major lineages within Clade B had gCF = 0%, with sCF values of 35.4–39.4%, only slightly above the random expectation of 33.3%. The node uniting *P. maculatus* with *P. assimilis* and *P. longicorpus* had gCF = 15.38% and sCF = 40.57%, despite a bootstrap support of 99.9%. In contrast, the well-supported nodes defining the *Ageneiogarra*, *Ptychidio*, *Semilabeo*, and *Discogobio* clades had both high bootstrap support and high concordance (gCF ≥ 92%, sCF ≥ 94%).

## 4. Discussion

### 4.1. Mitogenome Features of P. maculatus

The *P. maculatus* mitogenome shows no unusual structural or compositional features. Its gene content, gene arrangement (13 PCGs, 22 tRNAs, 2 rRNAs), AT-rich base composition and incomplete stop codons in several PCGs all match the teleost ground plan [41] including the two published congeners (*P. assimilis* and *P. longicorpus*) [19,20] and other Semilabeonini mitogenomes [43,44], indicating a structurally complete mitogenome consistent with the typical teleost organization. The D-loop was not characterized in the two congeneric reports [19,20]. In *P. maculatus*, it contains the full set of elements typical of fish D-loop (ETAS, central conserved and CSB domains), consistent with the homologous sequences examined, indicating that this architecture is conserved among the Semilabeonini species examined here rather than specific to *P. maculatus*. Tandem-repeat-driven control-region length variation has also been documented among closely related cyprinids; for example, the control regions of two *Dawkinsia* species differ substantially in length owing to a tandem repeat present in one species and absent in the other [45]. The (AT)n copy-number variation observed here (10–54 copies across Semilabeonini) is consistent with this general pattern.

A consistent feature of the *P. maculatus* mitogenome is the strand asymmetry in base composition: H-strand-encoded PCGs show negative GC-skew, whereas the L-strand-encoded *nad6* shows the opposite pattern (positive GC-skew and negative AT-skew). This pattern is consistently reported for *nad6* across fishes [39] and is most plausibly explained by strand-specific mutational bias during replication rather than by gene-specific selection. The pronounced AT-richness at the third codon positions (68.4% AT) and the strongly negative GC-skew at synonymous sites indicate relaxed constraint at synonymous positions, consistent with mutation pressure dominating over selection at fourfold-degenerate sites. Among H-strand PCGs, *nad2* and *atp8* show the highest AT-skews, but the basis of this variation remains unclear because comparable skew estimates are not available for the congeners.

### 4.2. Codon Usage and Selection Pressure

The strong bias toward A/U-ending codons in *P. maculatus* mitochondrial PCGs is consistent with the genome-wide AT-rich composition, as also reported in other teleost mitogenomes [41]. The predominant, though not exclusive, preference for A at the wobble position, exemplified by CGA, CUA, CCA, and UCA (RSCU > 2.0), may reflect mutational pressure favoring A/T nucleotides, and may be linked to strand asymmetries arising during replication. The exclusive use of UGA over UGG for tryptophan (RSCU = 1.83 vs. 0.17) corroborates the vertebrate mitochondrial genetic code. Such codon usage patterns may affect phylogenetic inference, as synonymous site saturation—particularly at third codon positions—is a recognized risk in mitogenomic datasets and can obscure deep phylogenetic signals in general. Xia’s test indicates that this general concern is not realized in the present dataset (Section 4.5).

The Ka/Ks ratios of all 13 PCGs were far below 1, indicating that the mitogenome of Semilabeonini is under purifying selection as a whole. This pattern is consistent with that reported for most fishes. Semilabeonini is mainly distributed in karst regions of southern China and Southeast Asia, with considerable variation in microhabitat use among lineages [6]. Some taxa (e.g., *Rectoris posehensis*) [46] inhabit fast-flowing riffles, whereas others (e.g., *Rectoris luxiensis*) [47] occupy caves, subterranean rivers, or deep pools, and some (e.g., *P. longicorpus*) [14] exhibit a winter-retreat and summer-emergence habit. Such seasonal migration across torrential and lentic, epigean and hypogean environments may be associated with distinct demands on energy metabolism. It is plausible that this ecological diversity is associated with the observed constraint, although this remains a hypothesis that the present data do not test.

Differences among genes may reflect the division of roles among complexes. cox genes were strongly constrained (*cox1* = 0.006, *cox3* = 0.010), with *cox1* showing the second-lowest Ka/Ks after *nad4L* (0.003); cytochrome c oxidase directly determines ATP yield, leaving little room for trial and error. Within nad genes, *nad4L* was extremely low (0.003), whereas *nad2*–*nad6* (0.025–0.054) were markedly higher than cox and *cytb*. Complex I is known to participate not only in electron transfer but also in reactive oxygen species (ROS) regulation and metabolic substrate flexibility, which provides a possible, though untested, context for the observed pattern. For species with a winter-retreat and summer-emergence habit, seasonal transitions between activity and dormancy involve large changes in metabolic rate. We hypothesize that complex I may require greater functional plasticity under fluctuating energy demand, which may be associated with the relatively relaxed constraint on some nad genes. In contrast, as a small subunit, the low Ka/Ks of *nad4L* may be related to its structural position or to tight interactions with other subunits, although we cannot exclude other explanations and this remains speculative. The relatively high Ka/Ks of *atp8* (0.044) is mainly attributable to its extremely low Ks (0.248, the lowest among all genes) rather than an elevated Ka (0.011). Given that *atp8* is among the shortest PCGs and has few synonymous sites, its elevated value is more likely a statistical artifact than a signal of relaxed selection, although we cannot fully exclude the latter. Because gene expression and metabolic activity were not measured in this study, the functional inferences in this section—including those linking Ka/Ks patterns to energy metabolism or to the winter-retreat and summer-emergence habit—remain hypothetical and require further testing.

### 4.3. Phylogenetic Placement of P. maculatus

The present mitogenomic analysis places *P. maculatus* within Semilabeonini: in the full PCG dataset it forms a clade with *P. assimilis* and *P. longicorpus*, within which it is the sister group to the latter two combined (BS = 99.9%, PP = 1). However, this placement is only provisionally resolved and conflicts with the first + second codon position tree: it receives low gene and site concordance (gCF = 15.38%, sCF = 40.57%) and when third codon positions are excluded *P. maculatus* is instead recovered as sister to *H. microstomata* (Figure S6; Section 3.5), a result that reflects the sensitivity of this placement to the exclusion of third-codon positions rather than a robust alternative hypothesis. The precise position of *P. maculatus* within *Parasinilabeo* is therefore not yet robustly resolved. Consequently, the monophyly of *Parasinilabeo* itself is recovered only in the full PCG dataset and is likewise not robust (Section 3.5); we therefore treat it as tentative, and the genus-level conclusion as tentatively resolved at best, pending broader taxon and nuclear-marker sampling. In the full PCG dataset, this result is consistent with the morphological assignment of the species to *Parasinilabeo* [12,13,14] and with earlier molecular studies that recovered the monophyly of *Parasinilabeo* [5,6,7,19,20]. This agreement is, however, only partial and does not extend to the first + second codon position analysis, and the genus-level conclusion therefore remains tentative. This caution is reinforced by the fact that oromandibular structures are pervasively homoplastic in this tribe [2,6,8,9] and thus provide only auxiliary support for generic limits.

*P. longicorpus* has been proposed as a possible synonym of *P. assimilis* on combined molecular and morphological grounds [7]. In the present analysis the two were treated as separate operational taxonomic units and provisionally recovered as sister groups. Their PCG-based distance is 7.47%, the lowest among the three *Parasinilabeo* species examined, but it still falls within the genus-wide interspecific range (7.47–9.62%; Table S6) and is far above the intraspecific values typically reported for fishes. This distance therefore does not support conspecific status. At the same time, a single mitochondrial distance is a coarse criterion, affected by among-lineage rate variation, divergence time and isolation history. The relatively high interspecific divergence within *Parasinilabeo* (7.47–9.62%) is consistent with its fragmented karst distribution and possibly with incomplete taxonomic resolution of the genus. We therefore leave the synonymy question open, to be tested with nuclear markers and population-level sampling.

Several *Parasinilabeo* species (e.g., *Parasinilabeo microps* [13], *P. longibarbus* [14] and *P. longiventralis* [15]) could not be included in the present analysis, because no complete mitogenome sequences are currently available in public databases. The relationships recovered here therefore rest on only three species and cannot yet represent the phylogenetic framework of *Parasinilabeo* as a whole. A robust test of the genus’s validity, however, awaits the inclusion of the missing species.

*Parasinilabeo* species are endemic to the karst regions of South China, distributed across the Yangtze and Pearl River drainages [10,11,12,13,14,15], whereas *P. maculatus* is currently known only from the Qiupu River in Shitai County, Anhui [12] and a few upper Ganjiang River tributaries [22]. This discrepancy, together with the phylogenetic distinctiveness of *P. maculatus*, may be consistent with a complex evolutionary history of the genus involving multiple independent colonization events or ancient vicariance within the karst landscape. However, testing these hypotheses would require divergence-time estimation, broader taxon sampling, and explicit biogeographic modeling. Although they are beyond the scope of the present study, they represent the most promising directions for future work on this genus.

### 4.4. Taxonomic Implications for Semilabeonini

Within Semilabeonini, non-monophyly was recovered for several genera, most notably *Sinocrossocheilus* and *Pseudocrossocheilus,* each represented here by only two species. *P. tridentis* was recovered as sister to *S. bamaensis*, whereas *S. labiatus* was distant from *S. bamaensis*, so that neither genus was recovered as monophyletic as currently defined. Because these inferences rest on only two species per genus and the deeper nodes within Clade B received weak support (BS < 70%, PP < 0.95), several alternative explanations cannot be excluded: mtDNA introgression following historical hybridization, misidentification or cryptic species among the sampled material, and stochastic or long-branch effects. The non-monophyly of these two genera is therefore regarded as preliminary. With this caveat, this pattern is nonetheless consistent with earlier findings that morphological characters traditionally used for generic delimitation—particularly oromandibular structures such as lips, barbels, and rostral folds—exhibit pervasive homoplasy within Labeoninae [6,7,8,9]. Previous studies have interpreted the repeated evolution of these structures as convergent adaptations to fast-flowing habitats, a hypothesis that has long been suspected to undermine morphology-based classifications. Our mitochondrial results provide additional tentative evidence of discordance between molecular relationships and morphology, but they do not independently demonstrate the evolutionary origin of these morphological characters. Formal taxonomic revision of these genera will require nuclear markers, morphological evidence, and broader taxon sampling.

Within Clade A, *Discogobio* was not recovered as monophyletic. In contrast to the sparsely sampled genera discussed above, this genus was represented by 14 species/lineages (Table 1). *Discocheilus wui* and *Discolabeo wuluoheensis* nested within the *Discogobio* clade. The genetic distances between *Discogobio* and *Discocheilus wui* (and between *Discogobio* and *Discolabeo wuluoheensis*) fell within the interspecific range of *Discogobio* (≤7.15%). Although this result is based solely on mitochondrial data, it rests on comparatively dense sampling and is therefore more robust than those for the two sparsely sampled genera. However, it still requires independent confirmation. This suggests that the generic status of these morphologically intermediate taxa deserves further investigation, particularly through broader taxon sampling and multi-locus data.

### 4.5. Phylogenetic Signal Quality and Causes of Low Resolution

Concordance factors calculated for the full-data ML tree (Figure 5a; Table S8) showed that several deep nodes within Clade B received low support in both ML and BI analyses (BS < 70%, PP < 0.95), and the position of *P. maculatus* changed when third codon positions were excluded (Figure S6). To assess whether this reflects methodological artifacts or limited signal, we examined substitution saturation and node concordance.

Xia’s test showed little saturation: Iss was significantly lower than Iss.c for the full dataset (Iss = 0.116 vs. Iss.c = 0.818/0.572), the first and second codon positions (Iss = 0.034), and even the third codon position (Iss = 0.317), all with *p* < 0.0001 (Table S7). Saturation is therefore not the primary cause of the low resolution.

Concordance factors were calculated for each node (Table S8). The deep nodes separating the major lineages within Clade B had gCF = 0%, indicating that none of the 13 single-gene trees supported them, and their sCF values (35.4–39.4%) were only slightly above the random expectation of 33.3%. The node uniting *P. maculatus* with *P. assimilis* and *P. longicorpus* had a gCF of only 15.38% and an sCF of 40.57%, with discordant gene trees mainly due to polyphyly (gDFP = 76.92%), despite a bootstrap support of 99.9%. In contrast, the well-supported nodes defining the *Ageneiogarra*, *Ptychidio*, *Semilabeo*, and *Discogobio* clades had both high bootstrap support and high concordance (gCF ≥ 92%, sCF ≥ 94%), confirming that these represent the well-resolved backbone of the tree.

Three factors likely explain the low resolution at deep nodes. First, the internal branches subtending these nodes are very short (0.0018–0.0035 substitutions/site in the BI tree), consistent with a rapid radiation in which few informative sites are available. Second, the strong purifying selection inferred from Ka/Ks (all PCGs ≪ 1) further reduces variable sites at deep divergences. Third, taxon sampling remains sparse for several genera (e.g., *Hongshuia*, *Pseudocrossocheilus*, *Sinocrossocheilus*), and incomplete lineage sorting or historical mitochondrial introgression may contribute additional conflict.

We therefore interpret the low support at deep nodes as a genuine reflection of limited signal and gene-tree conflict, rather than a methodological artifact. The placement of *P. maculatus* within Semilabeonini is consistently recovered, but its precise position is not robustly resolved by the present mitochondrial dataset and will require denser taxon sampling and nuclear markers.

### 4.6. Genetic Distance Anomalies and Their Interpretation

The exceptionally low interspecific distances detected among some *Discogobio* species (0.04–0.88%) and between *Semilabeo notabilis* and *S. obscurus* (0.35%) fall within the range typically expected for intraspecific variation in fishes [42]. Conversely, the exceptionally high intraspecific distance within *Discogobio macrophysallidos* (4.25%) exceeds the average interspecific value for the genus (4.10%). The broad distances (0.19–7.17%) among the three “uncertain species” warrant particular caution. These anomalies may be explained by several non-exclusive possibilities: recent divergence with incomplete lineage sorting [48]; taxonomic over-splitting due to subtle morphological differentiation; cryptic diversity warranting further investigation; or species misidentification. Among these, misidentification is a plausible but unconfirmed explanation, especially in morphologically similar groups or where voucher specimens cannot be examined. The most conspicuous cases are the 0.04% PCG distance between *Discogobio bismargaritus* PP384505 and *Discogobio laticeps* NC045918 and the 0.35% between *Semilabeo notabilis* NC045916 and *S. obscurus* MN229747 (Table S6). Values of this magnitude are typical of intraspecific rather than interspecific comparisons in fishes [42], so these pairs are the most likely candidates for misidentification or unrecognized conspecific status, and the corresponding vouchers and original records should be re-examined before further taxonomic or phylogenetic use. The possibility that the uncertain operational taxonomic units comprise multiple species should therefore be treated as a hypothesis requiring morphological and multilocus validation. Misidentification in open sequence repositories is well recognized [49,50], underscoring the need for voucher verification, taxonomic curation, and integrative taxonomy. A related issue has been raised in recent cyprinid mitogenomic work [45], in which the newly sequenced mitogenome of *Dawkinsia denisonii* differed from the published GenBank reference (AP011244) by 5.24% (881 SNPs and 22 InDels), a level of divergence interpreted as previously unrecognized intraspecific diversity rather than technical error. Taken together, these examples reinforce the need to verify publicly available mitochondrial sequences—and, where possible, their voucher specimens—before taxonomic reinterpretation.

### 4.7. Conservation Implications

*P. maculatus* is characterized by an extremely restricted distribution and low population densities. Local residents also reported a seasonal habitat shift, with winter retreat into caves and summer emergence into torrential streams, although this behavior was not directly observed or quantified in the present study. These traits render the species particularly vulnerable to habitat degradation and stochastic demographic processes. The present mitogenome, derived from a single specimen, represents a reference-level genetic resource. Analyses of population genetic diversity, genetic health, population structure, and conservation-unit delimitation require population-level sampling and are beyond the scope of this dataset. The sequence reported here instead provides a foundation for future studies. Given the logistical challenges of sampling this elusive species during its reportedly brief seasonal window, mitogenomic approaches offer an efficient means of generating the genetic data needed to support future conservation planning, including the design of captive breeding or translocation programs.

## 5. Conclusions

In this study, we sequenced and characterized the complete mitogenome of *P. maculatus* for the first time. It shows vertebrate-typical structure and base/codon usage consistent with other teleosts, and phylogenetic analysis places *P. maculatus* within Semilabeonini, where it is provisionally placed as sister to *P. assimilis* + *P. longicorpus* in the full PCG dataset. However, this placement is not robustly supported by gene and site concordance factors and conflicts with the first + second codon position tree, in which *P. maculatus* is sister to *H. microstomata*. The monophyly of *Parasinilabeo* is therefore tentatively resolved at best, and needs further verification with broader sampling and nuclear markers. Based on the present sampling, some genera of Semilabeonini were not recovered as monophyletic, and this also requires confirmation. As single-specimen data, this mitogenome is only a baseline reference; resolving relationships within *Parasinilabeo* and Semilabeonini and assessing genetic diversity or conservation units will require population-level and multilocus analyses.

## Figures and Tables

**Table 1:** List of species and 50 mitogenome sequences included in phylogenetic analysis.

No.	Species	Author	Accession	Size (bp)	AT (%)	AT-Skew	GC-Skew
1	*Ageneiogarra imberba*	(Garman, 1912)	NC025562	16,600	57.3	0.100	−0.247
2			MF958973	16,599	57.3	0.099	−0.248
3	*Cophecheilus bamen*	Zhu, Zhang, Zhang & Han, 2011	MK387703	16,588	58.2	0.110	−0.266
4	*Decorus decorus*	(Peters 1881)	NC26221	16,607	58.1	0.116	−0.274
5	*Decorus rendahli*	(Kimura, 1934)	NC28169	16,586	58.1	0.113	−0.270
6	*Discocheilus wui*	(Chen & Lan, 1992)	NC45066	16,589	58.8	0.095	−0.259
7	*Discogobio antethoracalis*	Zheng & Zhou, 2008	PQ789991	16,591	58.6	0.098	−0.258
8	*Discogobio bismargaritus*	Chu, Cui & Zhou, 1993	PP384505	16,601	58.6	0.102	−0.265
9			PQ789988	16,601	58.2	0.106	−0.266
10	*Discogobio brachyphysallidos*	Huang, 1989	NC83213	16,680	58.5	0.102	−0.263
11			OR486970	16,594	58.3	0.103	−0.263
12	*Discogobio elongatus*	Huang, 1989	PQ789989	16,605	58.5	0.105	−0.266
13	*Discogobio laticeps*	Chu, Cui & Zhou, 1993	NC45918	16,596	58.6	0.102	−0.265
14	*Discogobio longibarbatus*	Wu, 1977	NC36301	16,594	57.9	0.098	−0.254
15	*Discogobio macrophysallidos*	Huang, 1989	NC54299	16,593	58.0	0.103	−0.263
16			PQ464574	16,593	58.2	0.104	−0.262
17	*Discogobio propeanalis*	Zheng & Zhou, 2008	PQ789990	16,594	58.0	0.103	−0.262
18	*Discogobio* sp. ZF-11728	/	AP12151	16,594	58.2	0.106	−0.266
19	*Discogobio* sp. p FL-2023	/	OP994266	16,594	58.7	0.100	−0.262
20	*Discogobio* sp.	/	PQ464575	16,593	58.8	0.098	−0.261
21	*Discogobio tetrabarbatus*	Lin, 1931	NC24578	16,596	58.2	0.105	−0.264
22	*Discogobio yunnanensis*	(Regan, 1907)	NC25319	16,602	58.6	0.103	−0.267
23	*Discolabeo wuluoheensis*	Li, Lu & Mao, 1996	NC45067	16,587	58.7	0.094	−0.256
24	*Guigarra cailaoensis*	Wang, Chen & Zheng, 2022	OR492308	16,593	58.6	0.100	−0.254
25	*Hongshuia microstomata*	(Wang & Chen, 1989)	NC71769	16,673	58.0	0.103	−0.259
26	*Linichthys laticeps*	(Lin & Zhang, 1986)	NC82312	16,593	57.1	0.101	−0.255
27	*Paraqianlabeo lineatus*	Zhao, Sullivan, Zhang & Peng, 2014	NC63686	16,598	58.2	0.082	−0.237
28	*Parasinilabeo assimilis*	Wu & Yao, 1977	NC25947	16,602	58.4	0.107	−0.266
29	*Parasinilabeo longicorpus*	Zhang, 2000	NC53692	16,596	57.7	0.110	−0.262
30	*Parasinilabeo maculatus*	Zhang, 2000	PZ785808 *	16,606	58.7	0.094	−0.25
31	*Prolixicheilus longisulcus*	Zheng, Chen & Yang, 2010	NC50978	16,598	58.5	0.108	−0.267
32	*Pseudocrossocheilus liuchengensis*	(Liang, Liu & Wu, 1987)	NC45943	16,600	57.9	0.109	−0.267
33	*Pseudocrossocheilus tridentis*	(Cui & Chu, 1986)	NC56117	16,606	59.5	0.073	−0.228
34	*Pseudogyrinocheilus prochilus*	(Sauvage & Dabry de Thiersant, 1874)	NC24588	16,595	57.0	0.101	−0.247
35	*Ptychidio jordani*	Myers, 1930	KX151656	16,601	57.7	0.109	−0.268
36			NC24294	16,602	57.6	0.109	−0.268
37	*Ptychidio longibarbus*	Chen & Chen, 1989	PX23272	16,604	57.7	0.110	−0.270
38	*Ptychidio macrops*	Fang, 1981	MF457481	16,604	57.0	0.102	−0.259
39	*Rectoris luxiensis*	Wu & Yao, 1977	NC72181	16,592	58.2	0.069	−0.222
40	*Rectoris posehensis*	Lin, 1935	NC33390	16,594	58.0	0.107	−0.263
41	*Semilabeo notabilis*	Peters, 1881	NC45916	16,599	57.3	0.113	−0.265
42	*Semilabeo obscurus*	Lin, 1981	MN229747	16,598	57.3	0.113	−0.265
43			MN229748	16,598	57.3	0.112	−0.264
44			MN229749	16,599	57.3	0.113	−0.265
45			NC37408	16,598	57.3	0.112	−0.265
46	*Sinocrossocheilus bamaensis*	(Fang, 1981)	MF401443	16,594	58.6	0.085	−0.243
47			NC34789	16,594	58.7	0.084	−0.242
48	*Sinocrossocheilus labiatus*	Su, Yang & Cui, 2003	NC30247	16,603	59.5	0.089	−0.246
49	*Spinibarbus sinensis*	(Bleeker, 1871)	NC22465 ^#^	16,591	57.4	0.112	−0.253
50	*Tor sinensis*	Wu, 1977	NC22702 ^#^	16,579	56.8	0.123	−0.270

Note: * sequence obtained in this study; ^#^ outgroup; / no taxonomic authority available (undescribed or unidentified species). Multiple accessions under one species represent distinct sequences; see Section 2.5 for the removal of identical sequences.

**Table 2:** Composition and structure of the mitogenome of *P. maculatus*, comprising 37 genes and the control region (D-loop).

NO.	Gene	Strand	Position		Size	Intergenic Nucleotides	Codon	
			From	To			**Start**	**Stop**
1	*tRNA-Phe*	H	1	69	69	0		
2	*12S rRNA*	H	70	1024	955	0		
3	*tRNA-Val*	H	1025	1096	72	0		
4	*16S rRNA*	H	1097	2782	1686	0		
5	*tRNA-Leu* ^(UAA)^	H	2783	2858	76	0		
6	*nad1*	H	2859	3833	975	4	ATG	TAA
7	*tRNA-Ile*	H	3838	3909	72	−2		
8	*tRNA-Gln*	L	3908	3978	71	1		
9	*tRNA-Met*	H	3980	4048	69	0		
10	*nad2*	H	4049	5093	1045	0	ATG	T--
11	*tRNA-Trp*	H	5094	5164	71	2		
12	*tRNA-Ala*	L	5167	5235	69	1		
13	*tRNA-Asn*	L	5237	5309	73	33		
14	*tRNA-Cys*	L	5343	5408	66	1		
15	*tRNA-Tyr*	L	5410	5481	72	1		
16	*cox1*	H	5483	7033	1551	0	GTG	TAA
17	*tRNA-Ser* ^(UGA)^	L	7034	7104	71	3		
18	*tRNA-Asp*	H	7108	7179	72	14		
19	*cox2*	H	7194	7884	691	0	ATG	T--
20	*tRNA-Lys*	H	7885	7960	76	1		
21	*atp8*	H	7962	8126	165	−7	ATG	TAG
22	*atp6*	H	8120	8803	684	−1	ATG	TAA
23	*cox3*	H	8803	9587	785	0	ATG	TA-
24	*tRNA-Gly*	H	9588	9659	72	0		
25	*nad3*	H	9660	10,008	349	0	ATG	T--
26	*tRNA-Arg*	H	10,009	10,078	70	0		
27	*nad4L*	H	10,079	10,375	297	−7	ATG	TAA
28	*nad4*	H	10,369	11,749	1381	0	ATG	T--
29	*tRNA-His*	H	11,750	11,818	69	0		
30	*tRNA-Ser* ^(GCU)^	H	11,819	11,887	69	1		
31	*tRNA-Leu* ^(UAG)^	H	11,889	11,961	73	3		
32	*nad5*	H	11,965	13,788	1824	−4	ATG	TAA
33	*nad6*	L	13,785	14,306	522	0	ATG	TAA
34	*tRNA-Glu*	L	14,307	14,375	69	4		
35	*cytb*	H	14,380	15,520	1141	0	ATG	T--
36	*tRNA-Thr*	H	15,521	15,592	72	−1		
37	*tRNA-Pro*	L	15,592	15,661	70	0		
38	D-loop (control region)	H	15,662	16,606	945	0		

Note: H, heavy strand; L, light strand. T- and TA-- indicate incomplete stop codons, which are presumably completed by post-transcriptional polyadenylation. Positions of the D-loop elements (genome coordinates): ETAS domain, 15,662–15,905, including the TAS motif at 15,690–15,739; CD, 15,906–16,228, including CSB-F (15,906–15,932), CSB-E (15,958–15,982), CSB-D (15,998–16,021); CSB domain, 16,229–16,606, including CSB-1 (16,229–16,271), CSB-2 (16,350–16,372), CSB-3 (16,389–16,414) and the AT repeat region (16,469–16,494).

**Table 3:** Nucleotide composition and skewness values of *P. maculatus* mitogenome.

Regions	Strand	Size (bp)	T (U)	C	A	G	AT (%)	GC (%)	AT-Skew	GC-Skew
Full genome	H	16,606	26.6	25.8	32.1	15.5	58.7	41.3	0.094	−0.250
PCGs	all	11,403	29.0	26.1	30.0	14.9	59.0	41.0	0.017	−0.274
	H	10,881	28.4	26.8	30.8	14.1	59.2	40.9	0.041	−0.311
	L ^a^	522	43.1	11.7	14.0	31.2	57.1	42.9	−0.510	0.455
1st codon	all	3801	22.5	24.7	27.0	25.8	49.5	50.5	0.091	0.021
	H	3627	22.0	25.4	27.8	24.9	49.8	50.3	0.117	−0.01
	L ^a^	174	33.3	10.9	10.9	44.8	44.2	55.7	−0.506	0.608
2nd codon	all	3801	40.8	27.1	18.6	13.5	59.4	40.6	−0.374	−0.335
	H	3627	40.5	27.5	18.9	13.0	59.4	40.5	−0.363	−0.356
	L ^a^	174	46.0	19.5	10.9	23.6	56.9	43.1	−0.616	0.093
3rd codon	all	3801	23.9	26.4	44.5	5.3	68.4	31.7	0.302	−0.667
	H	3627	22.6	27.4	45.7	4.3	68.3	31.7	0.338	−0.729
	L ^a^	174	50.0	4.6	20.1	25.3	70.1	29.9	−0.426	0.692
*atp6*	H	684	30.0	26.5	31.0	12.6	61.0	39.1	0.017	−0.356
*atp8*		165	26.7	26.7	35.2	11.5	61.9	38.2	0.137	−0.397
*cox1*		1551	31.0	24.4	27.9	16.7	58.9	41.1	−0.054	−0.188
*cox2*		691	25.8	27.2	31.3	15.8	57.1	43.0	0.096	−0.266
*cox3*		785	28.2	27.4	28.5	15.9	56.7	43.3	0.007	−0.265
*cytb*		1141	30.4	25.6	30.4	13.6	60.8	39.2	0.000	−0.306
*nad1*		975	27.1	27.8	31.1	14.1	58.2	41.9	0.069	−0.328
*nad2*		1045	24.1	30.6	32.8	12.4	56.9	43.0	0.153	−0.422
*nad3*		349	34.1	24.6	27.8	13.5	61.9	38.1	−0.102	−0.293
*nad4*		1381	27.5	26.6	32.4	13.4	59.9	40.0	0.082	−0.331
*nad4L*		297	26.9	31.6	25.6	15.8	52.5	47.4	−0.026	−0.333
*nad5*		1824	28.6	26.0	32.7	12.7	61.3	38.7	0.066	−0.343
*16S rRNA*	H	1686	20.6	22.4	37.0	20.0	57.6	42.4	0.284	−0.056
*12S rRNA*		955	19.9	26.0	32.4	21.8	52.3	47.8	0.238	−0.088
tRNA	L/H	1563	27.2	20.7	29.2	22.9	56.4	43.6	0.035	0.050
D-loop	H	945	33.4	20.0	33.7	12.9	67.1	32.9	0.004	−0.216

Note: H, heavy strand; L, light strand. ^a^ L-strand data are from *nad6* gene.

**Table 4:** The relative synonymous codon usage (RSCU) of *P. maculatus* mitogenome.

Codon	Count	RSCU	Codon	Count	RSCU	Codon	Count	RSCU	Codon	Count	RSCU
UUU(F)	118	1.02	UCU(S)	37	0.96	UAU(Y)	64	1.13	UGU(C)	8	0.64
UUC(F)	113	0.98	UCC(S)	57	1.47	UAC(Y)	49	0.87	UGC(C)	17	1.36
UUA(L)	147	1.42	UCA(S)	83	2.15	UAA(*)	6	3.43	UGA(W)	109	1.83
UUG(L)	29	0.28	UCG(S)	7	0.18	UAG(*)	1	0.57	UGG(W)	10	0.17
CUU(L)	103	1.00	CCU(P)	32	0.60	CAU(H)	38	0.70	CGU(R)	14	0.74
CUC(L)	73	0.71	CCC(P)	56	1.06	CAC(H)	71	1.30	CGC(R)	9	0.47
CUA(L)	252	2.44	CCA(P)	119	2.25	CAA(Q)	92	1.88	CGA(R)	52	2.74
CUG(L)	16	0.15	CCG(P)	5	0.09	CAG(Q)	6	0.12	CGG(R)	1	0.05
AUU(I)	204	1.40	ACU(T)	49	0.64	AAU(N)	46	0.75	AGU(S)	12	0.31
AUC(I)	87	0.60	ACC(T)	105	1.37	AAC(N)	76	1.25	AGC(S)	36	0.93
AUA(M)	149	1.65	ACA(T)	142	1.85	AAA(K)	71	1.82	AGA(*)	0	0
AUG(M)	32	0.35	ACG(T)	11	0.14	AAG(K)	7	0.18	AGG(*)	0	0
GUU(V)	61	1.07	GCU(A)	56	0.68	GAU(D)	27	0.72	GGU(G)	38	0.62
GUC(V)	31	0.55	GCC(A)	131	1.60	GAC(D)	48	1.28	GGC(G)	44	0.72
GUA(V)	113	1.99	GCA(A)	135	1.65	GAA(E)	91	1.75	GGA(G)	130	2.11
GUG(V)	22	0.39	GCG(A)	6	0.07	GAG(E)	13	0.25	GGG(G)	34	0.55

The single capital letters in parentheses indicate the corresponding amino acids (one-letter code); * indicates stop codons. Total number of codons analysed (13 PCGs, stop codons included) = 3801; excluding stop codons = 3794.

## Data Availability

The original data presented in this study are openly available in the GenBank database at https://www.ncbi.nlm.nih.gov/genbank/ (accessed on 10 October 2026) under accession number PZ785808 (for the mitochondrial genome of *Parasinilabeo maculatus*). All other data are contained within the article and its Supplementary Materials.

## Supplementary Materials

The following supporting information can be downloaded at: https://www.mdpi.com/article/10.3390/ani16213447/s1, Table S1: The obtained next-generation sequencing data of *Parasinilabeo maculatus* in the study; Table S2: The Scaffold1 information for assembling *Parasinilabeo maculatus* mitogenome in the study; Table S3: Sequences of primers used for amplification of mitochondrial *cytb* and D-loop of *Parasinilabeo maculatus*; Table S4: Partitioning schemes and substitution models selected for maximum-likelihood (ML) and Bayesian inference (BI) analyses in this study; Table S5: Convergence diagnostics and 95% HPD intervals of parameters estimated in the Bayesian analysis; Table S6: The K2P genetic distance (%) between paired species within each genus of the family Semilabeonini; Table S7: Substitution saturation analysis (Xia’s test); Table S8: Gene concordance factors (gCF) and site concordance factors for key nodes; Figure S1: Predicted tRNA structures of mitogenome of *Parasinilabeo maculatus*; Figure S2: The structure and sequence of the control region (D-loop) of the mitogenome of *Parasinilabeo maculatus*; Figure S3: Sequence logos of the conserved motifs identified in the control region of the examined 48 Semilabeonini species; Figure S4: Predicted secondary structure of mitochondrial L-strand replication initiation region (OL) of *Parasinilabeo maculatus*; Figure S5: The scatter diagram for interspecific and intraspecific genetic distance of *Discogobio* (including *Discocheilus wui* and *Discolabeo wuluoheensis*) based on concatenated 13 PCGs of Semilabeonini; Figure S6: Maximum likelihood phylogenetic tree of Semilabeonini inferred from the first and second codon positions of the concatenated 13 PCGs, with node support values shown as SH-aLRT/ultrafast bootstrap (UFBoot) based on 1000 replicates.
