# Supplementary material for: Complete Mitochondrial Genome of the Poorly Known Fish Parasinilabeo maculatus (Cypriniformes: Cyprinidae) and Its Phylogenetic Implications
Source: Animals (Basel). 2026 Nov 1;16(21):3447. doi: 10.3390/ani16213447 (PMC13761638; doi:10.3390/ani16213447)
Supplement: Supplementary file 1 [file animals-16-03447-s001.zip › animals-4650529-supplementary.pdf]

Supplementary materials

1. Supplementary Tables:

Table S1. The obtained next-generation sequencing data of *Parasinilabeo maculatus* in the study

Raw data	Sample	Clean data	Sample
Total Reads Count	58831532	Total Reads Count	58606172
Total Bases Count (bp)	8824729800	Total Bases Count (bp)	8715759620
Average Read Length (bp)	150	Average Read Length (bp)	149
Q20 Bases Count (bp)	8669345227	Q20 Bases Count (bp)	8574747054
Q20 Bases Ratio (%)	98.24	Q20 Bases Ratio (%)	98.38
Q30 Bases Count (bp)	8555655159	Q30 Bases Count (bp)	8466486044
Q30 Bases Ratio (%)	96.95	Q30 Bases Ratio (%)	97.14
GC content (%)	38.33	GC content (%)	38.23
		Clean Reads (%)	99.62
		Low Quality (%)	0.33
		Too Many N (%)	0.00
		Too short (%)	0.05

Table S2. The Scaffold1 information for assembling *Parasinilabeo maculatus* mitogenome in the study

Parameter	Scaffold1
Length	16606
Covered_Reads_number	157046
Covered_Base_number	16281096
mean_depth	980.434542
Covered_percent	100
1X	16606
10X	16606
50X	16606
100X	16605
200X	16596

Table S3. Sequences of primers used for amplification of mitochondrial *cytb* and D-loop of *Parasinilabeo maculatus*

Sequence	Primer name	Primer sequences (5′—3′)	*T*a/℃
cytb	L14724	GACTTGAAAAACCACCGTTG	50
	H15915	CTCCGATCTCCGGATTACAAGAC	
D-loop	DL1	ACCCCTGGCTCCCAAAGC	55
	DH2	ATCTTAGCATCTTCAGTG	

Table S4. Partitioning schemes and substitution models selected for maximum-likelihood (ML) and Bayesian inference (BI) analyses in this study.

	Best partitions	Best model	
		ML	BI
P1	*atp6_codon1_atp8_codon3_nad2_codon1_nad3_codon1_nad4_codon1_nad5_codon1*	TIM2+F+I+G4	GTR+F+I+G4
P2	*atp6_codon2_cytb_codon2_nad2_codon2_nad3_codon2_nad4_codon2_nad5_codon2_nad6_codon2*	K3Pu+F+R3	GTR+F+I+G4
P3	*atp6_codon3_cox1_codon3_cox2_codon3_cox3_codon3_cytb_codon3_nad1_codon3_nad2_codon3_nad3_codon3_nad4L_codon3_nad4_codon3_nad5_codon3*	TIM2+F+I+G4	GTR+F+I+G4
P4	*atp8_codon1_cox2_codon1_cytb_codon1_nad1_codon1_nad4L_codon1_nad6_codon1*	TIM+I+G4	SYM+I+G4
P5	*atp8_codon2_cox1_codon2_cox2_codon2_cox3_codon2_nad1_codon2_nad4L_codon2*	TPM3u+F+I+G4	GTR+F+I+G4
P6	*cox1_codon1_cox3_codon1*	HKY+F+G4	SYM+I+G4
P7	*nad6_codon3*	TN+I+G4	HKY+F+G4

Table S5. Convergence diagnostics and 95% HPD intervals of parameters estimated in the Bayesian analysis.

Parameter	Mean	Variance	Lower	Upper	Median	minESS	avgESS	PSRF
TL{all}	2.539014	0.002338	2.437266	2.627758	2.538346	574.31	693.92	0.999
kappa{7}	13.141736	1.727328	10.701940	15.795110	13.035430	773.72	846.36	0.999
r(A<->C){1,2,3,4,5,6}	0.024527	0.000001	0.022501	0.026683	0.024543	554.30	556.56	1.001
r(A<->G){1,2,3,4,5,6}	0.447041	0.000108	0.427336	0.466134	0.446854	115.87	221.95	1.002
r(A<->T){1,2,3,4,5,6}	0.021133	0.000001	0.019124	0.023282	0.021091	346.97	393.88	1.000
r(C<->G){1,2,3,4,5,6}	0.007632	0.000001	0.005522	0.009987	0.007612	469.16	576.98	1.002

r(C<->T){1,2,3,4,5,6}	0.489702	0.000102	0.470800	0.508635	0.490138	203.55	269.90	1.002
r(G<->T){1,2,3,4,5,6}	0.009964	0.000002	0.007288	0.012552	0.009881	291.16	465.48	1.000
pi(A){all}	0.317412	0.000015	0.310266	0.325041	0.317443	422.69	462.08	0.999
pi(C){all}	0.289089	0.000011	0.282403	0.295467	0.289100	402.02	446.87	1.004
pi(G){all}	0.131027	0.000009	0.124699	0.136576	0.130943	308.30	336.92	1.002
pi(T){all}	0.262472	0.000011	0.256304	0.268865	0.262470	411.97	430.33	1.000
alpha{all}	1.195111	0.003232	1.085108	1.311184	1.195568	782.42	838.20	0.999
pinvar{1,2,3,4,5,6}	0.563568	0.000039	0.551047	0.575321	0.563603	856.00	864.68	1.000

Table S6. The K2P genetic distance (%) between paired species within each genus of the family Semilabeonini.

Genus	NO.	Species sequences	Genetic distance (%)																
			1	2															
Ageneiogarra	1	*A. imberba* NC025562																	
	2	*A. imberba* MF958973	0.18																
Cophecheilus	3	*C. bamen* MK387703																	
			4	5															
Decorus **3.61***	4	*D. decorus* NC026221																	
	5	*D. rendahli* NC028169	3.61																
			7	8	9	10	11	12	13	14	15	16	17	18	19	20	21	22	23
Discocheilus	6	*D. wui* NC045066	5.34	3.01	3.75	4.9	4.9	3.08	3.01	6.11	5.33	4.96	5.15	6.52	3.58	5.3	3.55	3.12	5.97
			7	8	9	10	11	12	13	14	15	16	17	18	19	20	21	22	
	7	*D. antethoracalis* PQ789991																	
Discogobio	8	*D. bismargaritus* PP384505	4.74																
**4.10***	9	*D. bismargaritus* PQ789988	5.32	2.81															
	10	*D. brachyphysallidos* NC083213	4.52	3.86	4.61														
	11	*D. brachyphysallidos* OR486970	4.52	3.86	4.61	0.00													

	12	*D. elongatus* PQ789989	4.46	1.97	2.62	3.87	3.87												
	13	*D. laticeps* NC045918	4.72	0.04	2.81	3.86	3.86	1.97											
	14	*D. longibarbatus* NC036301	5.59	5.32	5.84	4.36	4.36	5.16	5.32										
	15	*D. macrophysallidos* NC054299	4.87	4.53	5.05	3.66	3.66	4.38	4.53	4.37									
	16	*D. macrophysallidos* PQ464574	4.57	4.09	4.75	3.63	3.63	3.93	4.09	5.03	4.25								
	17	*D. propeanalis* PQ789990	4.66	4.14	4.89	2.93	2.93	4.11	4.14	4.71	3.93	4.04							
	18	*D.* sp. ZF-11728 AP012151	6.64	6.05	6.47	6.10	6.10	5.80	6.05	7.15	6.61	5.54	6.40						
	19	*D.* sp.p FL-2023 OP994266	5.24	2.72	1.34	4.46	4.46	2.61	2.72	5.72	4.93	4.62	4.83	6.55					
	20	*D.* sp. PQ464575	5.95	4.42	4.98	5.11	5.11	4.39	4.42	6.24	5.55	5.33	5.29	6.87	4.89				
	21	*D. tetrabarbatus* NC024578	5.21	2.65	1.32	4.42	4.42	2.57	2.65	5.68	4.87	4.56	4.75	6.46	0.19	4.91			
	22	*D. yunnanensis* NC025319	4.81	0.88	2.87	4.04	4.04	2.11	0.88	5.39	4.66	4.12	4.25	6.06	2.78	4.41	2.72		
Discolabeo			6	7	8	9	10	11	12	13	14	15	16	17	18	19	20	21	22
	23	*D. wuluoheensis* NC045067	5.97	5.68	5.33	5.97	4.87	4.87	5.16	5.30	6.31	5.45	5.13	5.28	7.17	5.79	6.08	5.85	5.32
Guigarra	24	*G. cailaoensis* OR492308																	
Hongshuia	25	*H. microstomata* NC071769																	
Linichthys	26	*L. laticeps* NC082312																	
Paraqianlabeo	27	*P. lineatus* NC063686																	
Parasinilabeo **8.77***			28	29	30														
	28	*P. assimilis* NC025947																	
	29	*P. longicorpus* NC053692	7.47																
	30	*P. maculatus* PZ785808	9.62	9.22															
Prolixicheilus	31	*P. longisulcus* NC050978																	
Pseudocrossocheilus **11.09***			32	33															
	32	*P. liuchengensis*																	
	33	*P. tridentis* NC056117	11.09																
Pseudogyrinocheilus	34	*P. prochilus* NC024588																	
Ptychidio **5.56***			35	36	37	38													
	35	*P. jordani* KX151656																	

	36	*P. jordani* NC024294	0.39				
	37	*P. longibarbus* PX023272	2.03	2.17			
	38	*P. macrops* MF457481	7.20	7.26	7.24		
			39	40			
Rectoris	39	*R. luxiensis* NC072181					
**10.44***	40	*R. posehensis* NC033390	10.44				
			41	42	43	44	45
	41	*S. notabilis* NC045916					
	42	*S. obscurus* MN229747	0.35				
Semilabeo	43	*S. obscurus* MN229748	0.22	0.36			
**0.23***	44	*S. obscurus* MN229749	0.04	0.37	0.24		
	45	*S. obscurus* NC037408	0.23	0.37	0.04	0.25	
			46	47	48		
Sinocrossocheilus	46	*S. bamaensis* MF401443					
**11.70***	47	*S. bamaensis* NC034789	0.61				
	48	*S. labiatus* NC030247	11.76	11.63			

Genus-level average values (*) were calculated as follows: (1) sequences with uncertain species names were excluded; (2) when multiple sequences were available for the same species, priority was given to the NC (reference) sequence, if available; and (3) as an exception, PQ789988 was used for *Discogobio bismargaritus*.

Table S7. Substitution saturation analysis (Xia's test)

Dataset	NumOTU	Iss	Iss.cSym	P	Iss.cAsym	P
All sites	32	0.116	0.818	0.0000	0.572	0.0000
Pos 1+2	32	0.034	0.815	0.0000	0.571	0.0000
Pos 3	32	0.317	0.809	0.0000	0.555	0.0000

Table S8. Gene concordance factors (gCF) and site concordance factors for key nodes

Bootstrap	gCF(%)	sCF(%)	Node
99.6/100	92.31	99.61	*Ageneiogarra clade*
100/100	100	99.06	*Ptychidio clade*
99.1/100	100	100	*Semilabeo clade*
100/100	100	100	*Discogobio bismargaritus* + *D. laticeps*
100/100	100	100	*Discogobio brachyphysallidos*
13.3/25	0	37.32	*Semilabeo* vs. (*Parasinilabeo* + *Hongshuia*)
35.5/20	0	39.43	*Guigarra* group vs. *Pseudocrossocheilus*/*Rectoris* group
85.4/29	0	36.72	*Guigarra group* vs. *Hongshuia*
60.6/57	7.69	35.84	*Rectoris luxiensis* + *R. posehensis*
79.9/54	0	35.42	Base of *Pseudocrossocheilus*/*Sinocrossocheilus*/*Ptychidio* clade
99.9/99	15.38	40.57	*P. maculatus* + (*P. assimilis*, *P. longicorpus*)

2. Supplementary Figures:

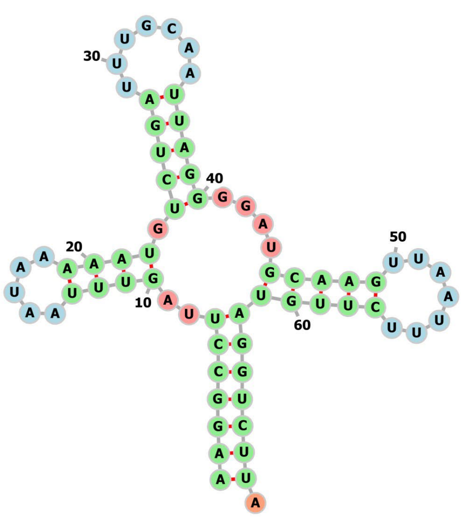

tRNA-Ala

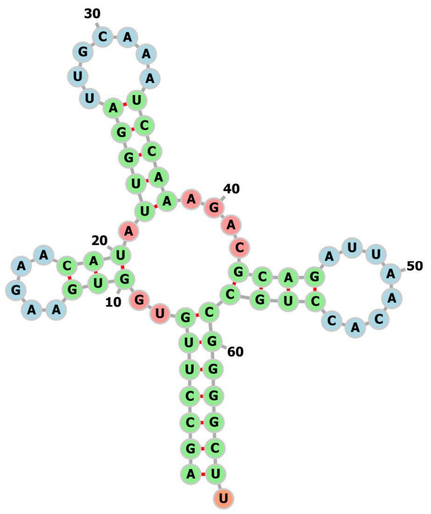

tRNA-Cys

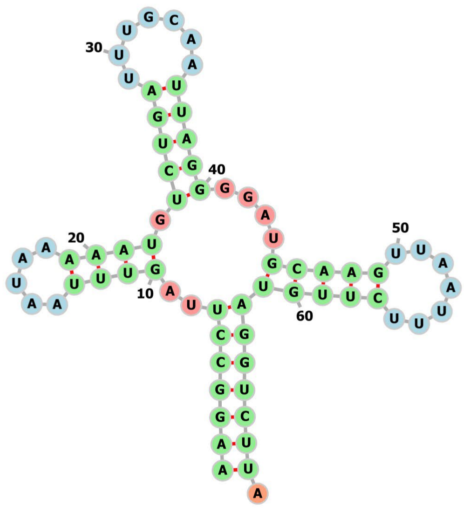

tRNA-Asp

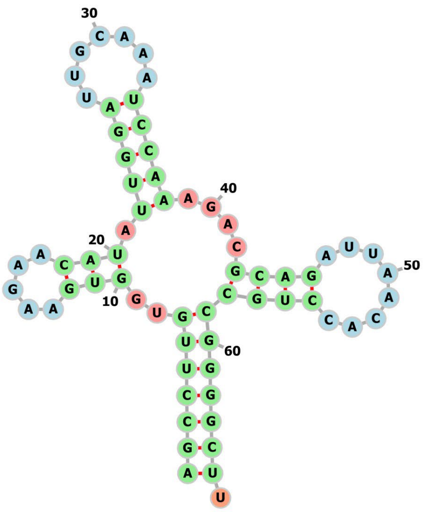

tRNA-Glu

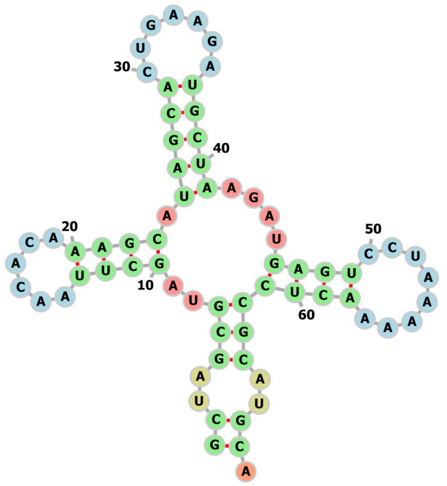

tRNA-Phe

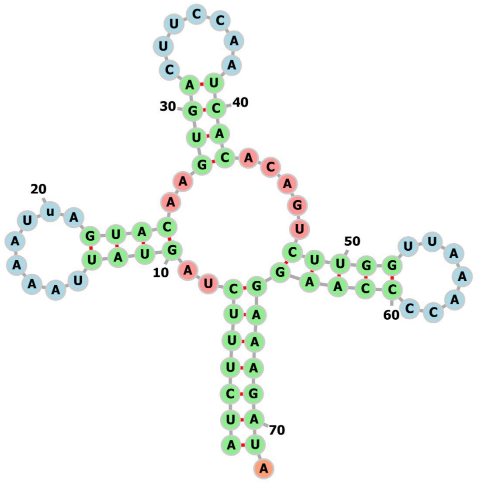

tRNA-Gly

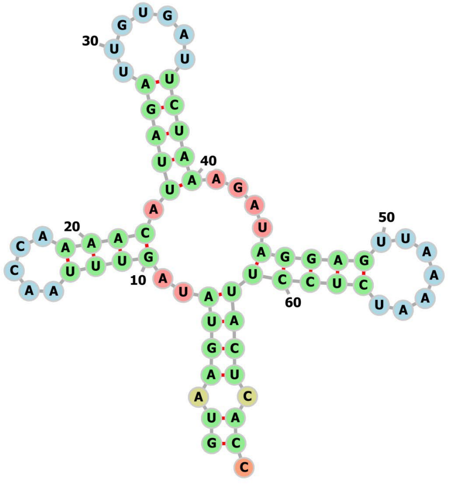

tRNA-His

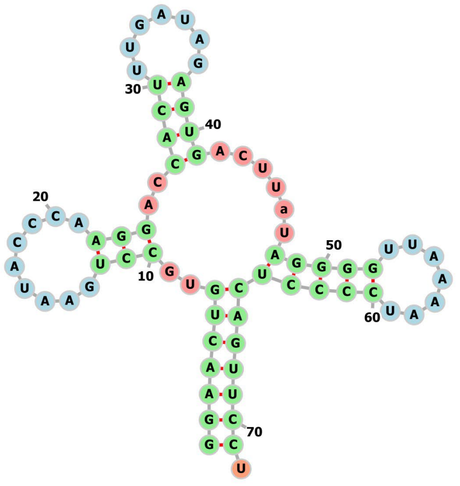

tRNA-Ile

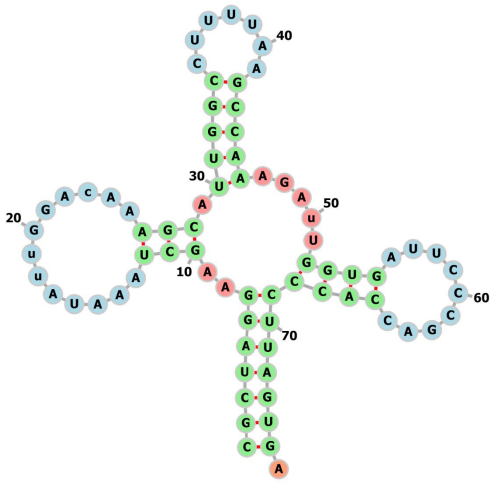

tRNA-Lys

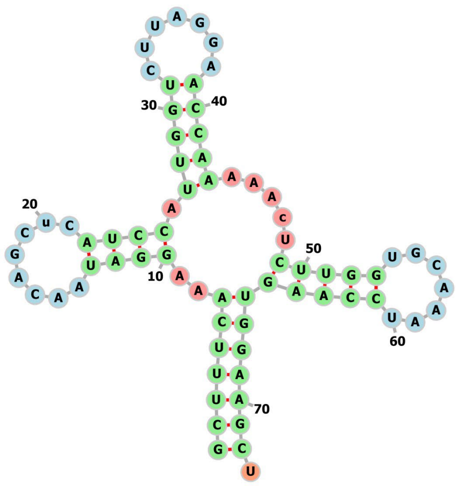

tRNA-Leu1(UAG)

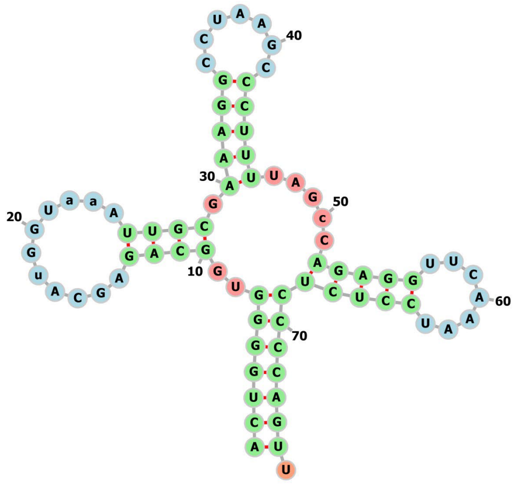

tRNA-Leu2(UAA)

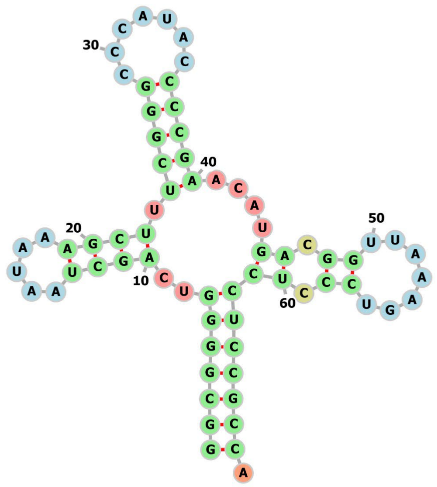

tRNA-Met

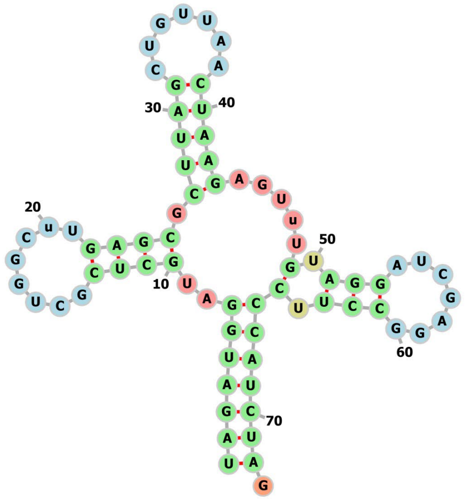

tRNA-Asn

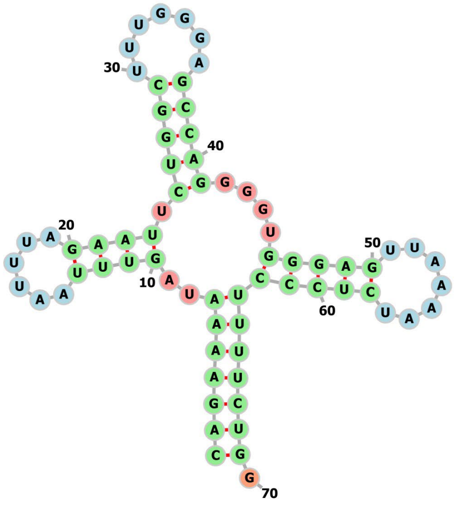

tRNA-Pro

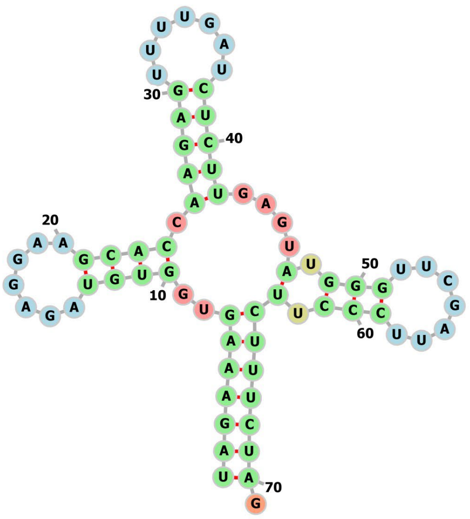

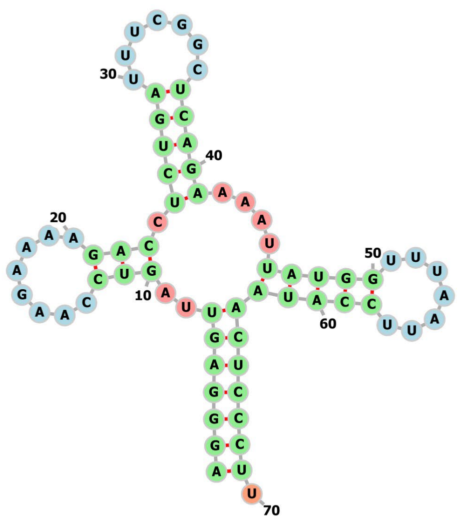

tRNA-Gln

tRNA-Arg

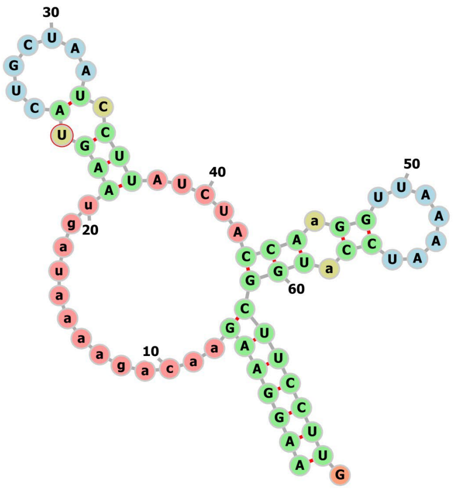

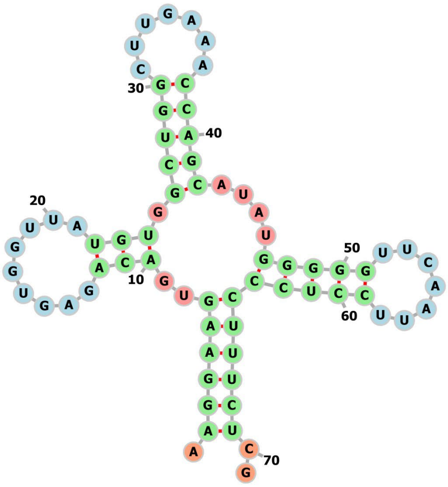

tRNA-Ser1(GCU)

tRNA-Ser2(UGA)

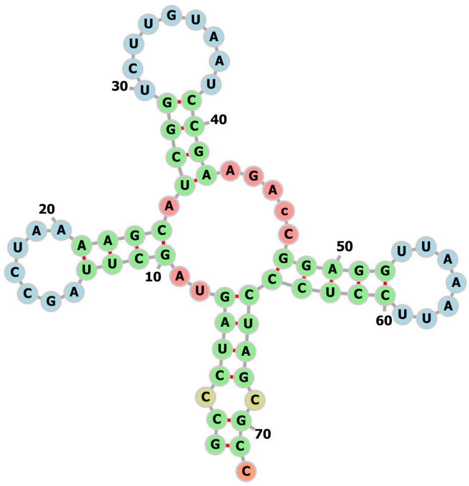

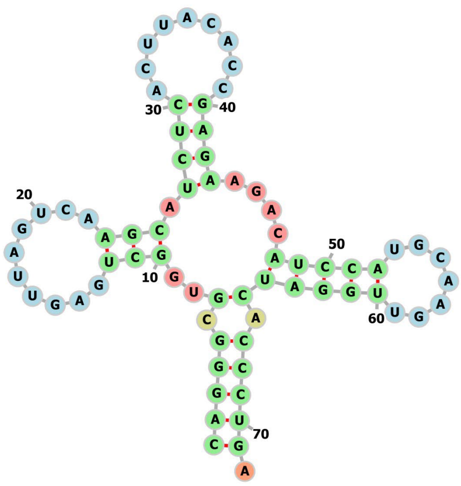

tRNA-Thr

tRNA-Val

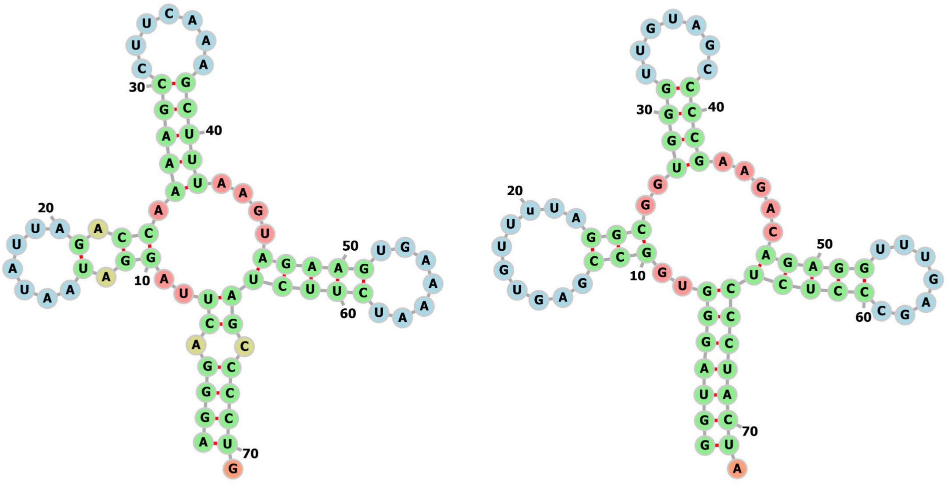

tRNA-Trp tRNA-Tyr

Figure S1. Predicted tRNA structures of mitogenome of *Parasinilabeo maculatus.*

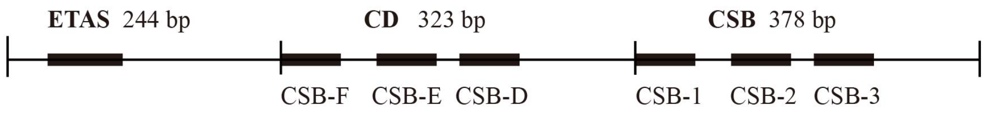

1 GGGTATAATC CACCCCTTTA TGGTTTAATA CATAATATGC TTGATTTTAC ATACATGTAT
TAS
61 TAATACATCT ATGTATTATC ACCAACTCAT TATTTTAACC ATAAAGCAAG TACTAATTTT
121 TAAGCTATTG CATAAGCATA TTATTAAGAC TACTATTTAA ACCTGGGAAA TAGATTATAC
181 CCCAAAAATT TGACCTCAAA TTTTTCCTTG AATAACTCAA CTATCATTTT AAACGTACAT
241 ATTAATGTAG TAAGAGACCA CCAACCAGTT TACATAAAGG CATATCATGC ATGATGGAAT
CSB-F
301 CAAGGACACA AATCGTGGGG GTAGCACAAT ATGAACTATT ACTGGCATCT GGTTCCTATT
CSB-E CSB-D
361 TCAGGAACAT AACTGTAATA TTTCCCCCCT CGGATAATTA TACTGGCATC TGATTAAGCT
421 AGTGGTGTGG TACATATGTT TCATTACCCA CCATGCGAGC ATCCCCTTAT ATGCATAACG
481 TTATTTTTTT TTGGTTTCCT TTCATCTTAC ATTTCAGAGT GCAAATATAA ACGTAAATCA
541 AGGTAGAACA TATTCCTTGC ATGTGTTAAT ATAAGTGAAT TATTATAAGA CATAACTTAA
CSB-1
601 GAATTACATA CTTCTAAGTC AAGTGCATAA CATATACATT CCTTATTCAA CTATACTTGC
661 TATTATGCCC CCTTTGGTTT TTACGCGTCA AACCCCCCTA CCCCCCTACG CCCAGTGAGT
CSB-2
721 CCTGTTCTCC TTGTCAAACC CCTAAACCAA GGAAGGCCCA AGAACGTATA AAGCCAACGA
CSB-3
781 GTTGAAATAT GAATTGGCTA TTCCATTATA TATATATATA TATATATATA TATGCATCAA
AT repeats
841 TTTTTACATT TTTACCGGTT TAATGTTAGC CTAATTAACC CTGCCAAAAA TCTGTAAAAA
901 TTTCTCGACA CTAAGTATTC TAATATAATA AATCA

Figure S2. The structure and sequence of the control region (D-loop) of the mitogenome of *Parasinilabeo maculatus.* The extended termination-associated sequence (ETAS) domain contains a termination-associated sequence (TAS). In TAS,

the core sequence (TACAT) and its reverse complementary sequence (ATGTA) are highlighted in yellow. The central conserved domain (CD) consists of the motifs CSB-F, E, D, and the conserved sequence block domain (CSB) contains CSB-1, 2, 3, respectively.

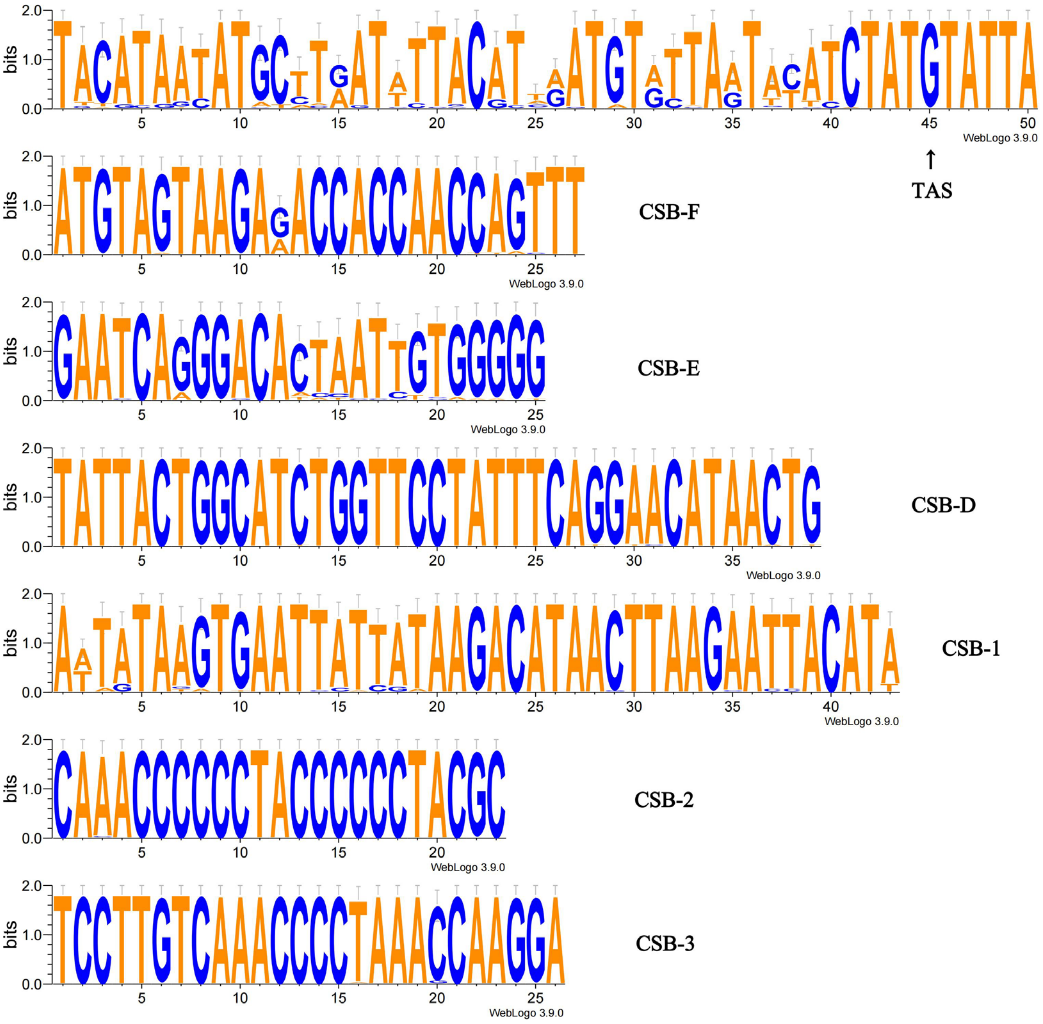

Figure S3. Sequence logos of the conserved motifs identified in the control region of the examined 48 Semilabeonini species. The height of each letter indicates the information content (bits) at that position; taller letters denote greater sequence conservation. The AT repeat region was excluded from this analysis because of variable copy numbers among species.

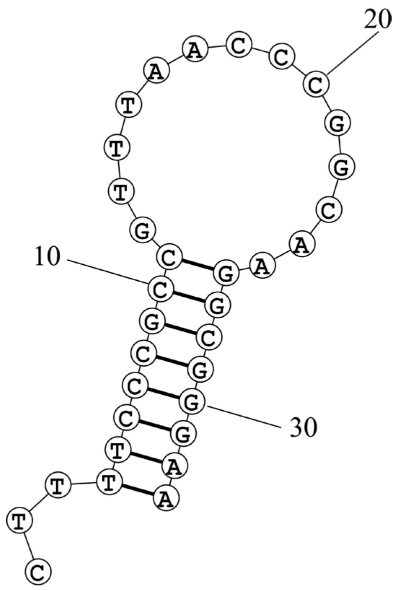

Figure S4. Predicted secondary structure of mitochondrial L-strand replication initiation region (OL) of *Parasinilabeo maculatus*. The structure features a stem-loop (hairpin) conformation, with a complementary stem region formed by Watson-Crick base pairing and a loop consisting of unpaired nucleotides. Numbers indicate nucleotide positions.

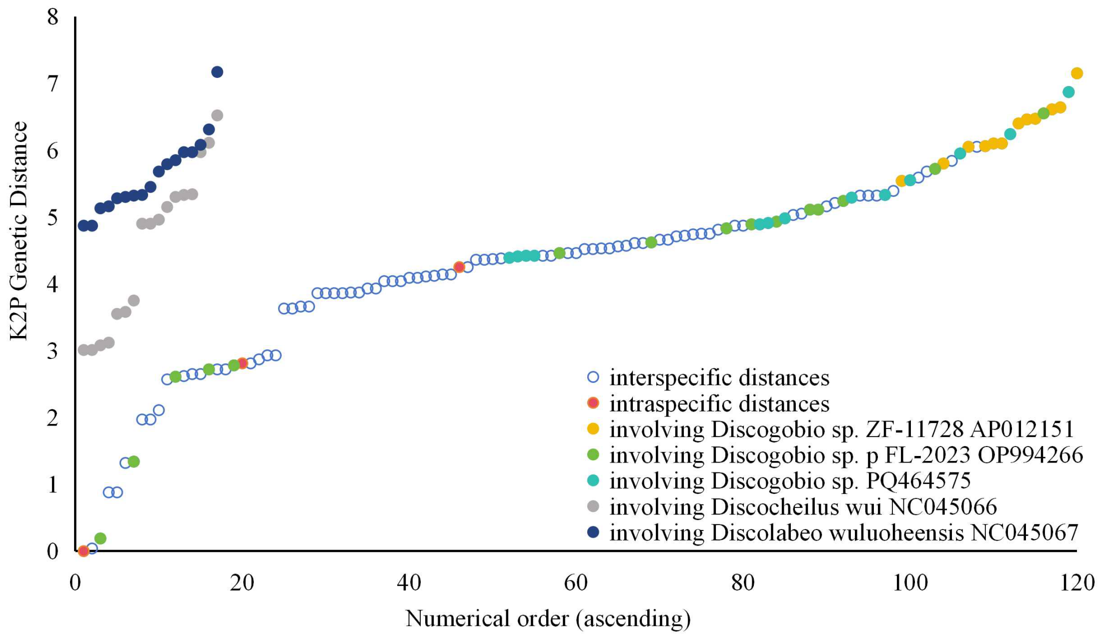

Figure S5. The scatter diagram for interspecific and intraspecific genetic distance of *Discogobio* (including *Discocheilus wui* and *Discolabeo wuluoheensis*) based on concatenated 13 PCGs of Semilabeonini.

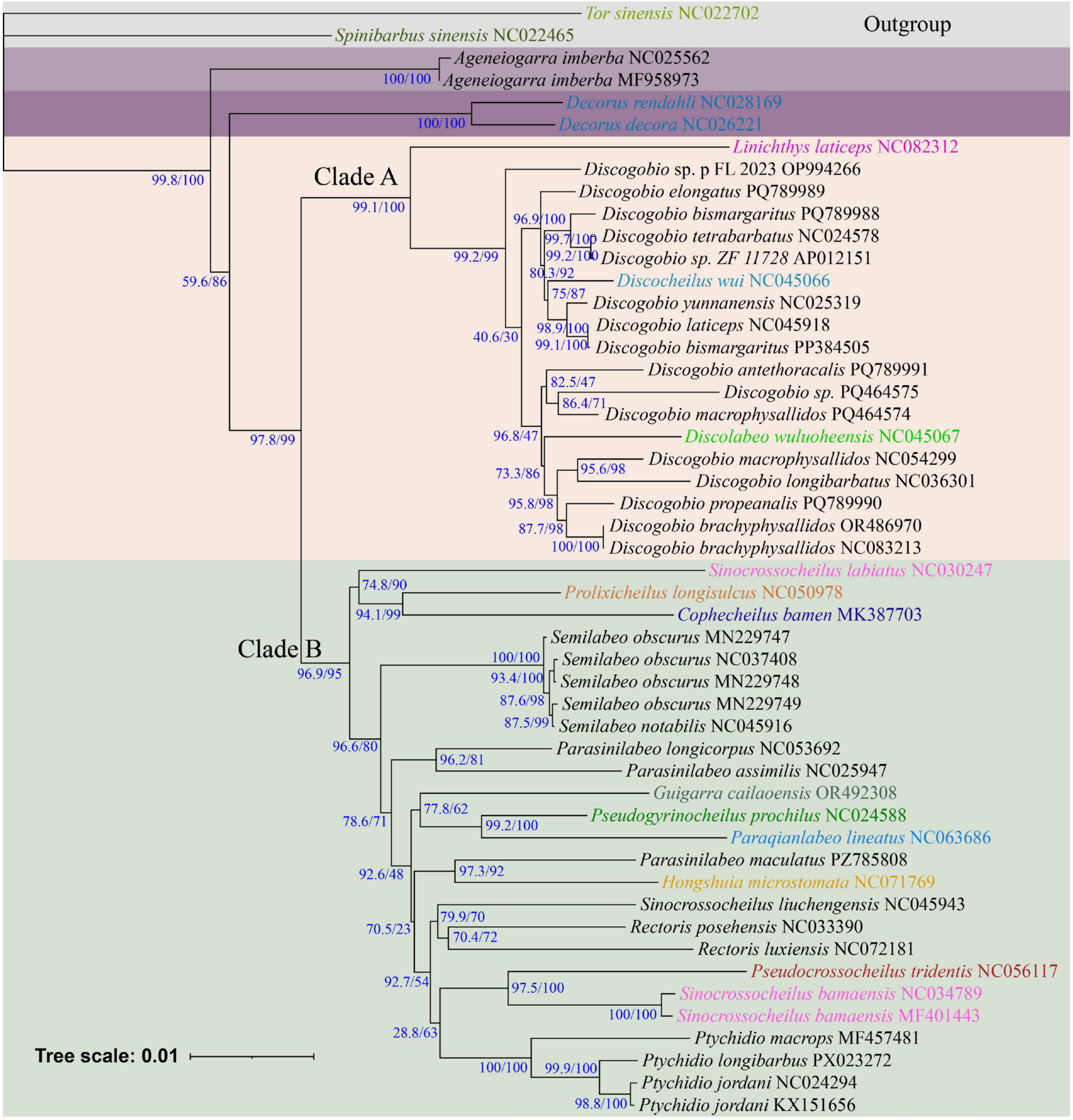

Figure S6. Maximum likelihood phylogenetic tree of Semilabeonini inferred from the first and second codon positions of the concatenated 13 PCGs, with node support values shown as SH-aLRT/ultrafast bootstrap (UFBoot) based on 1000 replicates.
